# Direct hOGG1-Myc interactions inhibit hOGG1 catalytic activity and recruit Myc to its promoters under oxidative stress

**DOI:** 10.1093/nar/gkac796

**Published:** 2022-09-26

**Authors:** Disha M Bangalore, Ingrid Tessmer

**Affiliations:** Rudolf Virchow Center, University of Würzburg, Josef Schneider Str. 2, 97080 Würzburg, Germany; Rudolf Virchow Center, University of Würzburg, Josef Schneider Str. 2, 97080 Würzburg, Germany

## Abstract

The base excision repair (BER) glycosylase hOGG1 (human oxoguanine glycosylase 1) is responsible for repairing oxidative lesions in the genome, in particular oxidised guanine bases (oxoG). In addition, a role of hOGG1 in transcription regulation by recruitment of various transcription factors has been reported. Here, we demonstrate direct interactions between hOGG1 and the medically important oncogene transcription factor Myc that is involved in transcription initiation of a large number of genes including inflammatory genes. Using single molecule atomic force microscopy (AFM), we reveal recruitment of Myc to its E-box promoter recognition sequence by hOGG1 specifically under oxidative stress conditions, and conformational changes in hOGG1-Myc complexes at oxoG lesions that suggest loading of Myc at oxoG lesions by hOGG1. Importantly, our data show suppression of hOGG1 catalytic activity in oxoG repair by Myc. Furthermore, mutational analyses implicate the C28 residue in hOGG1 in oxidation induced protein dimerisation and suggest a role of hOGG1 dimerisation under oxidising conditions in hOGG1-Myc interactions. From our data we develop a mechanistic model for Myc recruitment by hOGG1 under oxidising, inflammatory conditions, which may be responsible for the observed enhanced gene expression of Myc target genes.

## Graphical Abstract

**Graphical Abstract:**
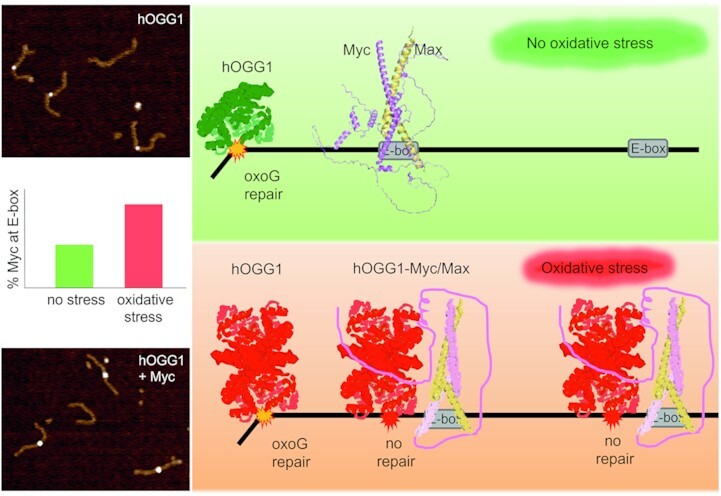
Oxidation induced hOGG1 dimerisation enhances interactions with the oncogene transcription factor Myc, which suppress DNA repair activity of hOGG1 and lead to loading of Myc on its Ebox binding motif.

## INTRODUCTION

Glycosylases are responsible for lesion search and recognition as well as excision of faulty bases *via* nucleophilic attack of the glycosidic bond (1) initiating base excision repair (BER). The abasic site product is then further processed by apurinic/apyrimidinic endonuclease (APE1 in human) as well as other BER proteins including DNA polymerase β. A number of glycosylases exist (at least 11 in humans) that are each specific for only a particular type or a few types of DNA modification. To rapidly detect their specific target lesions among a vast excess of non-damaged DNA bases, glycosylases bind non-specifically to non-damaged DNA and employ a combination of one-dimensional sliding along the DNA and three-dimensional hopping among DNA segments in their target site search (2–4). Finally, the enzymes employ a base flipping mechanism to specifically identify and excise their target (damaged DNA base) inside a catalytic pocket.

In addition, DNA repair unrelated functions of DNA glycosylases have been reported. Due to their low redox potential, guanine bases are most easily modified by reactive oxygen species (ROS) that arise, for example, during inflammatory stress conditions. Guanines have even been proposed to function as sinks for oxidation to protect downstream gene sequences from damage (5). Oxidation of guanines results in 7,8-dihydroguanine (oxoG) lesions that recruit the BER glycosylase hOGG1 (human oxo-guanine glycosylase 1). hOGG1 has been shown to modulate the expression of genes (6,7), in particular genes involved in the inflammatory response (8–10). Previous cellular studies have provided invaluable evidence for a role of oxoG lesions and hOGG1 in enhancing or reducing transcription of several different target genes of a range of transcription factors (5,10–22). These effects were correlated with transcription factor / hOGG1 co-localisation at promoters under oxidative stress conditions. Interestingly, this function appears to be specific for hOGG1 since other DNA glycosylases that also repair oxidative lesions in DNA did not induce transcription factor recruitment (18). In fact, the glycosylase NEIL2 even repressed NFκB binding to promoters (23).

Several models of hOGG1 interactions in transcription initiation have been proposed (6,8,12,13,16,18–20,24,25). These include both DNA repair dependent and independent mechanisms, DNA conformational modulation, and direct protein recruitment by hOGG1. A pivotal study also showed a function of the histone modifier lysine specific demethylase 1 (LSD1) in enhancement of gene transcription by the estrogen receptor transcription factor (11). LSD1 is a flavine adenine dinucleotide (FAD) dependent amine oxidase that demethylates histone H3 at positions K4 and K9, resulting in chromatin opening and a local burst of ROS (11,12). The authors proposed an intriguing model, in which ROS released during LSD1 histone demethylation activity locally oxidise the DNA leading to recruitment of hOGG1 to repair the induced oxoG lesions (11). APE1 displaces hOGG1 from the DNA after successful oxoG base removal by hOGG1 and induces a single strand cut, at which topoisomerase may load, which then eases DNA looping by locally relaxing the DNA. In their model, DNA looping enables interactions between transcription factors and RNA polymerase bound hundreds of base pairs (bp) apart on the DNA, to assemble the transcription machinery. Quantitative chromatin immunoprecipitation (qChip) studies were able to demonstrate LSD1 induced co-localisation of hOGG1 and the oncogene transcription factor c-Myc (referred to in the following simply as Myc) at transcription start sites and on E-box binding sequences within promoters of Myc activated genes (12). Importantly, subsequent enhancement of Myc-target gene expression by hOGG1 has been demonstrated (11,12). Myc plays an important role in tumour development and the activation of inflammatory processes (26). These studies also showed co-localisation of Myc and hOGG1 with the BER endonuclease APE1 and demonstrated that LSD1 and hOGG1 as well as APE1 were required for Myc transcription activation in their system (estrogen receptor transcription factor/Myc).

APE1 has also been shown to interact with transcription co-activators (27,28) and may thus itself serve to recruit the transcription machinery. These APE1 mediated protein interactions are central to another, highly interesting model of redox regulated gene transcription that is based on the induction of G quadruplex (G4) formation by hOGG1 repair activity (5,13,14,29,30). Stretches of guanines are often found in or near gene promoters and are particularly prominent targets for oxoG lesion formation. It has been shown that hOGG1 repair of ROS induced oxoG lesions in these G-tracks triggers G4 formation due to destabilisation of the DNA double helix structure by the resulting abasic site product (5,31). In this G4 model, APE1, whose catalytic activity is suppressed when bound to the abasic site (hOGG1 repair product) in the context of the G4 (13), recruits the transcription machinery by direct protein–protein interactions. Interestingly, these studies found that under oxidative stress, G4 formation in particular in the promoters of DNA repair genes may upregulate the levels of DNA repair proteins (such as NEIL1 or the BER polymerase β) to counteract the increasing amounts of induced DNA damages under these conditions (32).

Finally, an additional role of hOGG1 in inflammation has been reported, in which oxoG that is released from the DNA by hOGG1 repair activity activate RAS GTPase that then mediates increasing ROS levels in cells to further stimulate the immune response by activating inflammatory gene transcription (15,33,34). Again, this function seems to be specific for hOGG1, because other oxidative lesion glycosylases (e.g. NEIL1) did not induce this response (15).

All these studies established oxidation of guanines in DNA as an epigenetic mark (13,21). However, the mechanism of transcription regulation by oxoG lesions and hOGG1 is still unclear. Different mechanisms may exist depending on the sequence context of the gene promoter. In this context, up- as well as down-regulation of gene transcription by hOGG1 repair of oxoG lesions that led to G4 formation has been observed, and has been shown to depend on oxoG position (distance from promoter as well as DNA strand) (30). Furthermore, while the models involving LSD1 activity or G4 formation implied the necessity for oxoG repair by hOGG1 for transcription activation, hOGG1 catalytic activity was not required for enhancing pro-inflammatory gene expression (e.g. by NFκB) in other studies (10,16,18).

Here, we used atomic force microscopy (AFM) imaging to resolve the mechanistic details of hOGG1 interactions in transcription factor recruitment to their promoters at the molecular level (on the example system of Myc) under controlled, oxidising or reducing conditions. Oxidation of hOGG1 itself has been proposed to inactivate the enzyme while not affecting its DNA binding (35), to allow inactive, oxidised hOGG1 to recruit transcription factors such as Myc in the absence of oxoG repair functions by hOGG1. We demonstrate here that oxidation of hOGG1 leads to its dimerisation, but not inactivation. We further show that oxidation of hOGG1 modulates direct interactions with Myc (as well as LSD1). Importantly, we demonstrate recruitment of Myc to its E-box binding site in gene promoters by hOGG1 independent of hOGG1 catalytic activity and specifically under oxidising conditions. Furthermore, AFM structural comparison of hOGG1-DNA complex conformations in the absence and presence of Myc shows depletion of the lesion excision competent hOGG1 conformation at oxoG sites by Myc, as also supported by functional assays that demonstrate complete suppression of hOGG1 repair activity by Myc.

Such interplay between DNA repair and transcription provides an attractive model for the cellular response to DNA damage by upregulating the expression of proteins involved in the necessary steps for cell survival. On the other hand, Myc induced enhancement of inflammatory gene expression that further augments oxidative stress inside the cell likely represents a pathological process that occurs in particular in tumour cells with high levels of Myc as well as oxidative stress. Our data suggest the hOGG1-Myc interaction as a potential target for inhibitor development against Myc induced tumour progression.

## MATERIALS AND METHODS

### Proteins

hOGG1 was amplified from cDNA (Dharmacon) for SLIC cloning into pETM14 vector. Single amino acid mutants hOGG1 K249Q, C28A, C241A and C253A were generated from the wildtype hOGG1 gene by PCR and confirmed by sequencing. All primers are given in Supplementary Table S1. hOGG1 wildtype and mutants (in pETM14 vector) and Max (in pGEX4T1 vector) were expressed in *Escherichia coli* BL21(DE3) cells. LSD1 (in pCDF-Duet1 vector) was expressed in *E. coli* Rosetta (DE3) cells. hOGG1 and LSD1 were purified by Ni^2+^-NTA affinity chromatography followed by anion exchange chromatography (MonoQ 10/100 GL) and size exclusion chromatography (Superdex 200 16/600 GL). hOGG1 wildtype and mutants were eluted and stored in 20 mM Tris–HCl, pH 7.5, 200 mM NaCl, 5% glycerol, 0.5 mM DTT; LSD1 in 50 mM HEPES, pH 8.0, 150 mM NaCl, 5% glycerol, 0.5 mM DTT. Max was purified in a single step by glutathione sepharose bead affinity chromatography and eluted and stored in 20 mM HEPES, pH 7.9, 250 mM NaCl, 5% glycerol, as previously described (36). All proteins were purified to > 95% homogeneity as judged from Coomassie stained SDS-PAGE (polyacrylamide gel electrophoresis, Supplementary Figure S1A) and stored at -80°C until use. Concentrations of purified proteins were determined spectrophotometrically using extinction coefficients of 68 400 M^−1^cm^−1^ for his_6_-hOGG1, 80 800 M^−1^ cm^−1^ for his_6_-LSD1, and 26 400 M^−1^ cm^−1^ for GST-Max. All proteins were N-terminally tagged and tags were removed *via* protease digestion where required (see fluorescence polarisation assays). Purified GST tagged full length c-Myc was purchased from Antibodies.com.

### DNA

#### EMSA, fluorescence polarisation and hOGG1 activity assays

48 base pair (bp) double stranded DNA substrates were prepared by annealing respective top (oxoG-containing or undamaged) and bottom strands at 1:1 molar ratio. The bottom strand further contained a 5‘ Cy3 or AlexaFluor 647 (AF647) label for fluorescence detection. A list of all oligonucleotides (Sigma Aldrich) is shown in Supplementary Table S2.

#### AFM studies

DNA substrates for AFM were prepared from circular pUC19N plasmid (37) with a length of 2,729 bp as previously described (38,39) (for details see Supplementary Figure S2). To prepare the DNA substrate containing an E-box motif (CACGTG) or an oxoG lesion as well as E-box motif placed 8 nt apart, the E-box sequence was cloned into pUC19N plasmid (for primers see Supplementary Table S3) and successful insertion of the E-box was confirmed by sequencing. The final DNA substrates had a length of 505 bp and contained in their center the feature of interest, either oxoG (at 49.8% of DNA length), or oxoG and an E-box (at 49.8% and 51.8% DNA length, respectively), or only the E-box sequence (at 51.8% DNA length).

### Oxidising and reducing conditions

Samples for AFM experiments were incubated in AFM buffer (25 mM HEPES pH 7.5, 25 mM NaCl, 10 mM Mg_2_Cl_2_). For all other assays, samples were incubated in interaction buffer (20 mM Tris–HCl pH 7.6, 200 mM NaCl), which for SDS-PAGE (hOGG1 dimerisation assay and Western blots) further contained 5% glycerol. Experiments on protein interactions in the absence of DNA were performed in interaction buffer containing either 5 μM H_2_O_2_ or 5 mM DTT for oxidising or reducing conditions, respectively. For incubations under oxidative conditions involving DNA (EMSAs, activity assays, and AFM studies) and for Western blots, hOGG1 was pre-oxidised (after purification, prior to experiments), to avoid oxidative damage induction in the DNA by H_2_O_2_ in the reaction buffer. hOGG1 (5 μM) was pre-oxidised in incubation buffer containing 5 μM H_2_O_2_ for 3 h at 4°C, dialysed overnight against H_2_O_2_-free buffer at 4°C, concentrated, and flash frozen and stored in incubation buffer at –80°C until use. Incubations of protein-DNA samples were then carried out in H_2_O_2_-free and DTT-free buffer for oxidising conditions. For experiments with DNA under reducing conditions, native (non-oxidised) hOGG1 was used and incubations were carried out in buffer containing 5 mM DTT. All experiments were carried out at least in triplicate.

### Fluorescence polarisation

For assessing hOGG1-DNA binding affinities, 5 nM AF647 labelled oxoG-containing or undamaged 48 bp DNA substrates were incubated with increasing concentrations of hOGG1.

To quantify protein interactions by hOGG1, his_6_ tagged hOGG1 was labelled with Ni-NTA AlexaFluor 488 (AF488) dye by incubation at 1:3 hOGG1:dye ratio in interaction buffer for 30 min in the dark at ambient temperature. Excess dye was removed by repeated spin filtering using a 10 kDa MWCO filter for 10 min at 10 000 × g and 4°C. Using photospectrometry to determine the concentrations of AF488 and hOGG1 (with extinction coefficients ε_280nm_ = 68 400 M^−1^cm^−1^ for hOGG1 and ε_488nm_ = 73 000 M^−1^cm^−1^ for AF488), a ratio of dye:protein of ∼1 was obtained. To measure hOGG1 dimerisation, 5 nM AF488 labelled hOGG1 was then incubated with increasing concentrations of unlabelled hOGG1 (0.24 nM–8 μM). To investigate hOGG1 interactions with Myc or LSD1, 5 nM AF488 labelled hOGG1 was incubated with increasing concentrations of the unlabelled proteins (1.22 nM–2 μM for Myc and 1.38 nM–60 μM for LSD1) in interaction buffer.

All measurements were performed at 25°C under reducing as well as under oxidising conditions. Polarisation values were recorded using a CLARIOstar^R^ microplate reader (BMG Labtech) and polarisation curves were fit by single Hill equations with *R*^2^ ≥ 0.95 using Origin Pro software. Average binding affinities were calculated based on triplicate measurements.

### SDS-PAGE studies of hOGG1 dimerisation

hOGG1 was diluted to 5 μM in interaction buffer containing 5% glycerol and either oxidised by addition of 5 μM H_2_O_2_ or reduced by addition of 5 mM DTT to the buffer. In addition, oxidized hOGG1 was subsequently incubated with 5 mM DTT to test reversibility of hOGG1 oxidation. Samples were then denatured at 95°C in non-reducing SDS buffer and run on a 15% SDS polyacrylamide gel. Gels were visualised using a camera based imaging system (Fusion FX6, Vilber).

### Western blots of hOGG1 wildtype and mutants

Samples of 8 μM purified wildtype hOGG1, hOGG1_C28A_, hOGG1_C241A_ and hOGG1_C253A_ were either oxidised or reduced (see above, Oxidising and reducing conditions) and run on a non-reducing 15% SDS-PAGE gel. Gels were transferred to a nitrocellulose membrane in transfer buffer (25 mM Tris/HCl pH 8.3 190 mM glycine, 20% ethanol) using a Mini-transblot cell (Biorad) at 300 mA and 4°C for 1 h. Membrane blocking was performed using TBS (50 mM Tris/HCl pH 7.5 150 mM NaCl)-Albumin Fraction V 2.5% (w/v) followed by washing with TBS-T (50 mM Tris/HCl pH 7.5, 150 mM NaCl, 0.05% (w/v) Tween-20). The membrane was incubated overnight with the primary, mouse anti-His antibody **(**Sigma-Aldrich) at 4°C (diluted 1000× following manufacturer's instructions), followed by incubation with the secondary goat anti-mouse IgG antibody (diluted 10 000× in TBS). Blots were developed using Pierce™ ECL Western blotting substrate (ThermoFisher Scientific) and protein bands were visualised by chemiluminescence detection in the Vilber imaging system (see above).

### Electrophoretic mobility shift assays

Incubations of hOGG1 (300 nM), or hOGG1 and Myc (300 nM each) with DNA were carried out under oxidising or reducing conditions at room temperature for 30 min. 10 μl samples were loaded onto 15% native PAGE gels (with 29:1 acrylamide: bisacrylamide). Gels were run in 0.5× Tris–borate–EDTA (TBE) at 4°C and visualised using a camera based imaging system (Vilber, see above).

### hOGG1 activity assay

Pre-oxidised or reduced hOGG1 (150 nM–2 μM) was incubated with oxoG lesion DNA substrate (20 nM) for 30 min at 37°C (see Oxidising and reducing conditions above for buffer). For experiments on suppression of hOGG1 repair activity by Myc, hOGG1-oxoG DNA (300 nM hOGG1, 20 nM oxoG DNA) samples containing increasing concentrations of Myc (50–300 nM) were incubated for 30 min at 37°C. The abasic site generated by hOGG1 oxoG repair activity was then cleaved by incubation with 0.5 N NaOH for 10 min at ambient temperature and the reaction was stopped by heating to 95°C for 10 min, mixed with urea containing loading buffer (8 M urea) and loaded onto 15% polyacrylamide (37.5:1 acrylamide: bisacrylamide) 7 M urea gels. Gels were run in 0.5× TBE at 4°C and 100 V and visualised in the Fusion FX6 Vilber imaging system. Background corrected intensities of cleaved products and uncut bands were quantified in ImageJ.

### AFM imaging

For AFM experiments on hOGG1 ± Myc and oxoG containing DNA, hOGG1 wildtype or hOGG1_K249Q_ (150 nM) were incubated with oxoG lesion DNA (3 nM) in the absence or presence of Myc (30 nM) for 15 min at ambient temperature in AFM buffer (see above, Oxidising and reducing conditions). For experiments at reducing conditions, incubations were carried out in AFM buffer containing reducing agent (5 mM DTT). For experiments at oxidising conditions, pre-oxidised hOGG1 (wildtype or mutant) was used.

For experiments on Myc recruitment by hOGG1 to its E-box binding site in the presence of an upstream oxoG lesion, quantum dot (QD) conjugation of Myc was carried out as previously described for other proteins (39,40). Briefly, GST tagged Myc was incubated with mouse anti-GST monoclonal antibody (Thermo Fisher Scientific) in a 1:1 molar ratio at room temperature for 30 min (1 μM concentration). This mixture was then incubated with QD705 carrying F(ab') goat anti-mouse IgG secondary antibody (Thermo Fisher Scientific) for 30 min at 1:1 ratio (500 nM). Next, QD-labeled Myc was incubated with Max at 1:1 molar ratio and a concentration of 20 nM for 10 min to form a stable complex. For experiments, 2 nM QD-Myc/Max was incubated with 3 nM oxoG E-box DNA substrate in the absence or presence of 150 nM hOGG1 as described above. As controls, QD-Myc was incubated with oxoG E-box DNA in the absence of Max or hOGG1, and QD-Myc/Max with or without hOGG1 was incubated with DNA containing an E-box sequence but no oxoG lesion or an oxoG lesion but no E-box.

Samples were deposited onto freshly stripped mica, rinsed with filtered, deionised water, and dried in a stream of nitrogen for AFM imaging. Imaging was performed in air at a scan speed of 2.5 μm/s using a Molecular Force Probe (MFP) 3D (Asylum Research, Oxford Instruments) and AC240 imaging probes (Olympus) with nominal resonance frequency of ∼70 kHz and spring constant of ∼2 N/m.

### AFM data analysis

For experiments on hOGG1 and Myc on oxoG DNA (with oxoG at ∼50% (49.8%) of DNA substrate length), protein complex positions and respective DNA bend angles were determined using the extended automated MatLab DNA bend angle analysis tool (available at Open Science Framework https://osf.io/76e9s/) as previously described (41,42). Since the two DNA ends cannot be distinguished in our samples, position distributions are plotted to 50% DNA length. Protein complex volumes were measured using Image SXM (S. Barrett, University of Liverpool) as previously described (38,42), Data were plotted as histograms and fit by single or multiple Gaussians using Origin Pro software to obtain binding specificities to the oxoG lesion, DNA bend angle states and volumes of complexes from the centres of the Gaussian fits. Specificities *S* for oxoG binding were obtained as described (43) from the ratio of occupancy at the specific site (oxoG) defined by the area under the Gaussian A_sp_ at 50% DNA length and that of nonspecific DNA sites (*A*_nsp_, area of background from 0 to 50% of total DNA length) using $S\ = \ N\ ( {\frac{{Asp}}{{Ansp}}} ) + 1$, where N is the number of total available binding sites (number of base pairs, *N* = 505 bp). Binding frequencies at the oxoG lesion were determined from the number of binding events at the 50% position (bin 45–50% DNA length) per DNA in the images and are reported as averages (± standard deviations, SD) from triplicate experiments.

Positions of QD-labeled Myc were manually measured using the free hand line tool in ImageJ to determine the distance of QD centers from the closer DNA end as well as complete DNA substrate lengths. QDs were identified in the images based on heights, using a selection criterion of ≥3 nm (Supplementary Figure S3A) given by the average heights of ∼1.5 nm for protein complexes that did not contain QDs (hOGG1, Max) and ∼4.5 nm for QD-containing complexes (QD-Myc, QD-Myc/Max, or QD-Myc/Max + hOGG1). QD-Myc positions were plotted as histograms to obtain the frequency of Myc binding at the central oxoG-E-box position (45–50% DNA length, averages ± SD from triplicate experiments). In our DNA substrates, the oxoG lesion is located at 49.8%, the E-box at 51.8% (corresponding to 48.2% DNA length in our plots to 50% DNA length). As controls, we also performed experiments using DNA substrates that contained either only the oxoG lesion or only the E-box.

### Statistical analyses

Significances were calculated using a two-tailed student *t* test with * *P* < 0.05, ** *P* < 0.01, *** *P* < 0.005. *t* values are listed in Supplementary Table S4 for four degrees of freedom in all experiments (comparison of sets of triplicates).

## RESULTS AND DISCUSSION

### hOGG1 interacts directly with Myc and LSD1

**Figure 1.**
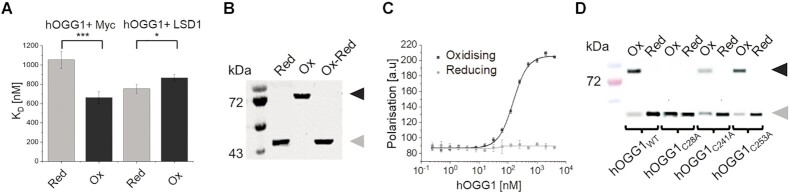
**hOGG1 interacts directly with Myc and undergoes dimerisation upon oxidation**. (**A**) Fluorescence polarisation measurements under reducing (grey) and oxidising (black) conditions show *K*_D_ values of 1055 ± 89 nM and 662 ± 65 nM for hOGG1–Myc interactions and 754 ± 47 nM and 867 ± 34 nM for hOGG1–LSD1 interactions, respectively. For details on significances see Supplementary Table S4. Data curves are shown in Supplementary Figure S5. Identical results for hOGG1 interactions with full length Myc and with the isolated N-terminal domain of Myc (Supplementary Figure S5A) implicate exclusively the N-terminal part of Myc in the interaction. (**B**) Oxidation of hOGG1 is reversible. Non-reducing SDS-PAGE reveals monomeric hOGG1 under reducing conditions (Red) and dimeric hOGG1 under oxidising conditions (Ox). Oxidised hOGG1 reverts to the monomeric state upon treatment of the oxidised protein with reducing conditions (Ox-Red). (**C**) Fluorescence polarisation measurements show strong hOGG1 dimerisation under oxidising conditions in the high nanomolar range, and absence of dimer formation under reducing conditions (see also SDS-PAGE in Supplementary Figure S6). (**D**) Western blots of hOGG1 variants, in which the three accessible cysteines (Supplementary Figure S1) have been mutated to alanines, implicate in particular hOGG1 C28 in disulfide bond formation for hOGG1 dimerisation under oxidising conditions. C241 and C253 partially contribute to hOGG1 dimerisation. Arrows indicate the dimer (black) and monomer (grey, *M*_r_ ∼40 kDa) form of hOGG1.

Using an *in vitro* pulldown assay, we found direct interactions between recombinantly expressed and purified hOGG1 and Myc under reducing as well as oxidising conditions (Supplementary Figure S4). The assay also showed direct interactions between LSD1 and Myc, as previously reported (12). Interestingly, fluorescence polarisation studies showed that oxidation of hOGG1 resulted in significantly enhanced affinities between hOGG1 and Myc (Figure 1A, *K*_D_ of ∼660 nM *versus* ∼1.1 μM for hOGG1/Myc under reducing conditions, see also Supplementary Figure S5). In contrast, the interaction between hOGG1 and LSD1 was slightly, but significantly decreased under oxidising conditions (Figure 1A, K_D_ of ∼870 nM *versus* ∼750 nM under reducing conditions). Oxidative stress characterises inflammatory conditions, for example in tumours, in which Myc expression is enhanced to concentrations in the low micromolar range (44) that thus support interactions with hOGG1, in particular under oxidising conditions.

### hOGG1 dimerises under oxidative conditions

To understand the above differences in hOGG1 interactions, we characterised the effects of oxidation on hOGG1. Non-reducing SDS gel and fluorescence polarisation analyses distinctly showed hOGG1 dimerisation in the high nanomolar range under oxidative conditions (Figure 1B,C and Supplementary Figure S6). In contrast, no dimer formation was observed under reducing conditions. Nuclear concentrations of hOGG1 have been reported to be ∼300 nM (45), sufficient to support hOGG1 dimerisation under oxidising conditions *in vivo*. Moreover, hOGG1 protein expression has been shown to be upregulated by factors between ∼2 and 7 in cancer tissues (46,47). Importantly, hOGG1 dimerisation was reversible in our assays when the oxidised protein was subsequently re-introduced into reducing environment (buffer containing 5 mM DTT, Figure 1B). However, dimers of oxidised hOGG1 were not disrupted by glutathione concentrations of up to ∼3 mM (Supplementary Figure S7), indicating high stability and physiological relevance of the dimer in particular under oxidative stress condition with glutathione depletion to ≤1 mM in the nucleus (48,49).

Mutations of the individual accessible cysteines in the protein (C28A, C241A, and C253A) implicate in particular the C28 residue in hOGG1 in dimerisation (Figure 1D and Supplementary Figure S1). Our data suggest that dimerisation may be at least partially mediated by disulfide bond formation between the C28 residues of hOGG1, for which no function could previously be assigned (50). Mutation of the C28 residue in hOGG1 to alanine also abrogated enhancement of Myc-hOGG1 affinities under oxidising conditions (Supplementary Figure S1D), further supporting a role of the hOGG1 dimer in interactions with Myc.

**Figure 2.**
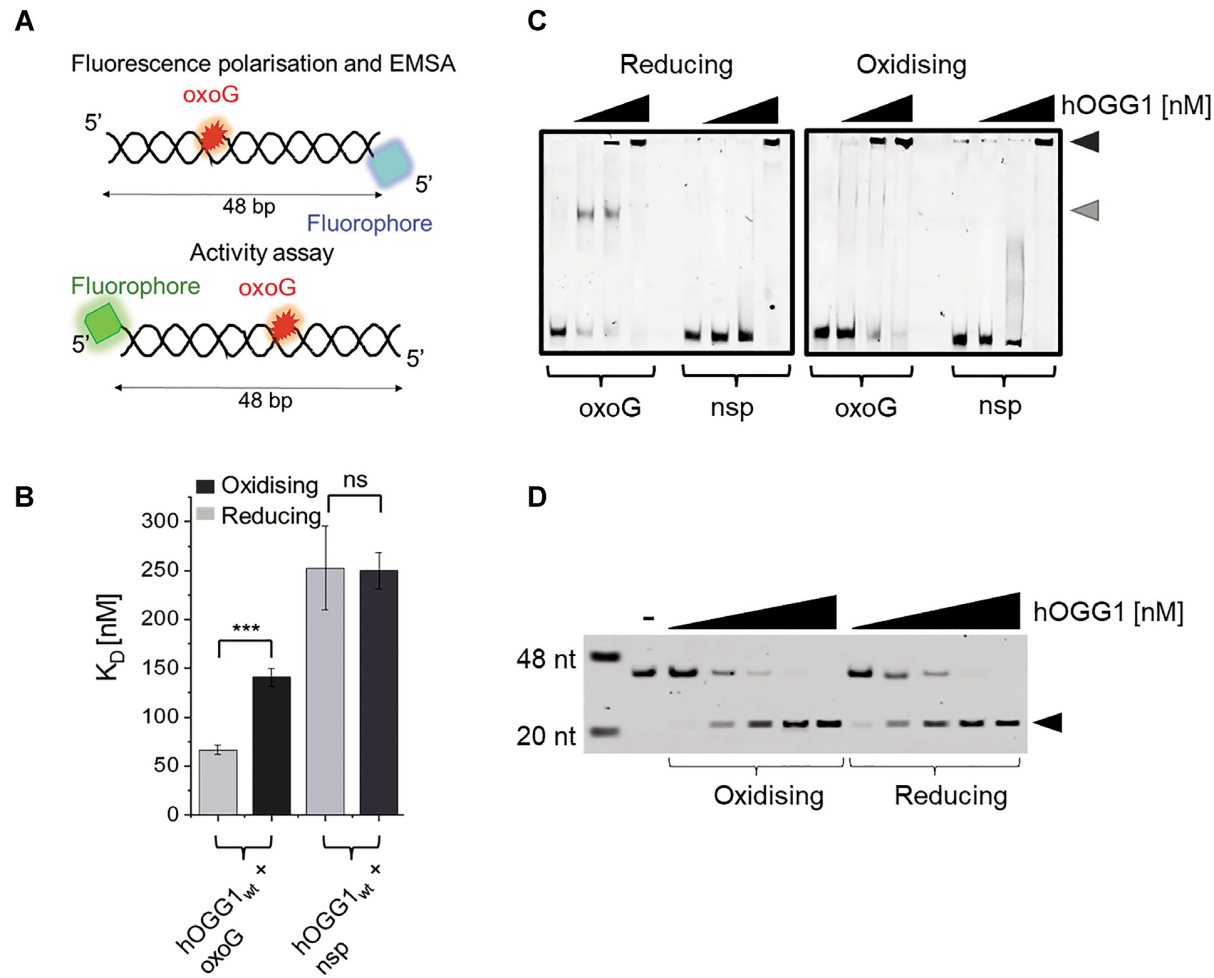
**Oxidation of hOGG1 affects its DNA binding but not catalytic activity**. (**A**) Schematic of the oxoG containing DNA substrates. The substrate for fluorescence polarisation and EMSA experiments contained an oxoG lesion at the 10^th^ position in the top strand and a fluorophore attached to the 5‘ end of the bottom strand. The DNA substrate for the activity assay contains the oxoG lesion at the 26^th^ position and the fluorophore was attached at the 5’end of the top, oxoG containing strand. (**B**) Fluorescence polarisation measurements under reducing (grey) and oxidising (black) conditions for hOGG1–oxoG interactions provided *K*_D_ values of 66 ± 5 nM and 140 ± 9 nM, respectively. *K*_D_’s of hOGG1 interactions with undamaged (non-specific, nsp) DNA were comparable for reducing and oxidising conditions (252 ± 43 nM and 250 ± 18 nM, respectively; ns = not significant). For details on significances see Supplementary Table S4. Binding curves are shown in Supplementary Figure S8. (**C**) EMSA analyses with titrations of hOGG1 (0, 150, 300 and 1000 nM) to oxoG-containing or non-specific DNA showed monomeric hOGG1 complexes exclusively for oxoG lesion DNA and under reducing conditions (grey arrow). At higher concentrations (300–1000 nM), hOGG1 formed higher order oligomers (presumably dimers) on the DNA (black arrow). Under oxidising conditions (right), stable binding to the oxoG substrate also required higher hOGG1 concentrations (300 nM versus 150 nM for reducing condition, left). Binding to the non-specific DNA substrate was independent of redox conditions and occurred exclusively as dimers and at higher concentrations than with oxoG-DNA, consistent with lesion recognition by hOGG1. (**D**) In hOGG1 activity assays, hOGG1 (0–1000 nM) demonstrated comparable oxoG repair under oxidising and reducing conditions (cleaved product is indicated by the arrow).

Consistent with hOGG1 repair activity in natively reducing environment inside the cell nucleus, binding of hOGG1 to its oxoG target lesions was significantly stronger under reducing compared to oxidising conditions (*K*_D_’s of ∼66 nM for hOGG1 in the presence of reducing agent *versus* ∼140 nM using non-reducing reaction conditions and pre-oxidised hOGG1, compared to ∼250 nM for undamaged DNA under both reducing and oxidising conditions, see Figure 2A,B, Table 1, and Supplementary Figure S8). Electrophoretic mobility shift assays (EMSAs) confirmed that oxidised hOGG1 binds to oxoG lesions as well as to undamaged DNA as a dimer (or higher order oligomer) while under reducing conditions a monomeric hOGG1 complex is formed at the oxoG lesion (Figure 2C). Interestingly, however, in contrast to previous speculations (6), oxidation/dimerisation did not affect oxoG repair activity by hOGG1 (Figure 2A, D).

**Table 1.** Binding to oxoG lesions in DNA

	Redox condition	Binding at oxoG * [counts/DNA]	SD	*K*_D_** (nM)	SD (nM)
hOGG1	Oxidising	0.10	0.004	140	9
	Reducing	0.18	0.06	66	5
hOGG1-Myc	Oxidising	0.17	0.0001	nd	nd
	Reducing	0.17	0.01	nd	nd
hOGG1_K249Q_	Oxidising	0.09	0.002	95	5
	Reducing	0.18	0.03	42	3
hOGG1_C28A_	Oxidising	0.14	0.01	61	11
	Reducing	nd	nd	65	8
hOGG1_K249Q_–Myc	Oxidising	0.10	0.01	nd	nd
	Reducing	0.15	0.004	nd	nd
hOGG1-Myc/Max	Oxidising	0.19	0.004	nd	nd
	Reducing	0.12	0.01	nd	nd
hOGG1_C28A_-Myc	Oxidising	0.14	0.01	nd	nd

[hOGG1] 150 nM, [Myc] 30 nM, [Max] 30 nM (where present).

nd: not determined; *from AFM positional analyses; **from fluorescence polarisation.

SD standard deviation from triplicate experiments (duplicates for hOGG1_K249Q_ ± Myc under oxidising/reducing conditions and hOGG1_C28A_ without Myc).

Interestingly, previous studies had also reported dimer formation by the cancer mutant hOGG1 S326C, which led to reduced binding affinity and specificity for its oxoG target lesions in DNA (51,52), as seen here for the wildtype dimer. However, dimeric S236C hOGG1 showed suppressed oxoG repair activity in these studies, while oxidation-induced dimerisation of wildtype hOGG1 did not inhibit hOGG1 catalytic activity (Figure 2D). These differences can be explained by the very different dimerisation interfaces for the wildtype protein and the S326C cancer variant. Dimerisation of hOGG1 *via* cysteines at position 326 interferes with oxoG processing by the enzyme. In contrast, the C28 dimerisation site is located in the N-terminus, far removed from the catalytic centre of hOGG1. Consistently, previous studies had shown no effect on hOGG1 repair activity by mutation of C28 to serine (50). Surprisingly, wildtype-like repair activity for C28S hOGG1 contrasted with strongly decreased oxoG binding affinity in these studies. This observation has been explained by indirect, long-range DNA interactions by the (dynamic) N-terminal domain of hOGG1 (50). Although mutation of C28 to alanine did not affect oxoG binding in our studies (Supplementary Figure S1B and E and Table 1, in contrast to mutation to serine (50)), dimerisation of wildtype hOGG1 at the N-terminal domain (*via* C28 residues) shows the same effect of reduced oxoG binding but unaffected catalytic activity (compared to monomers under reducing conditions).

### Myc induces conformational changes in hOGG1-oxoG complexes and suppresses hOGG1 catalytic activity

**Figure 3.**
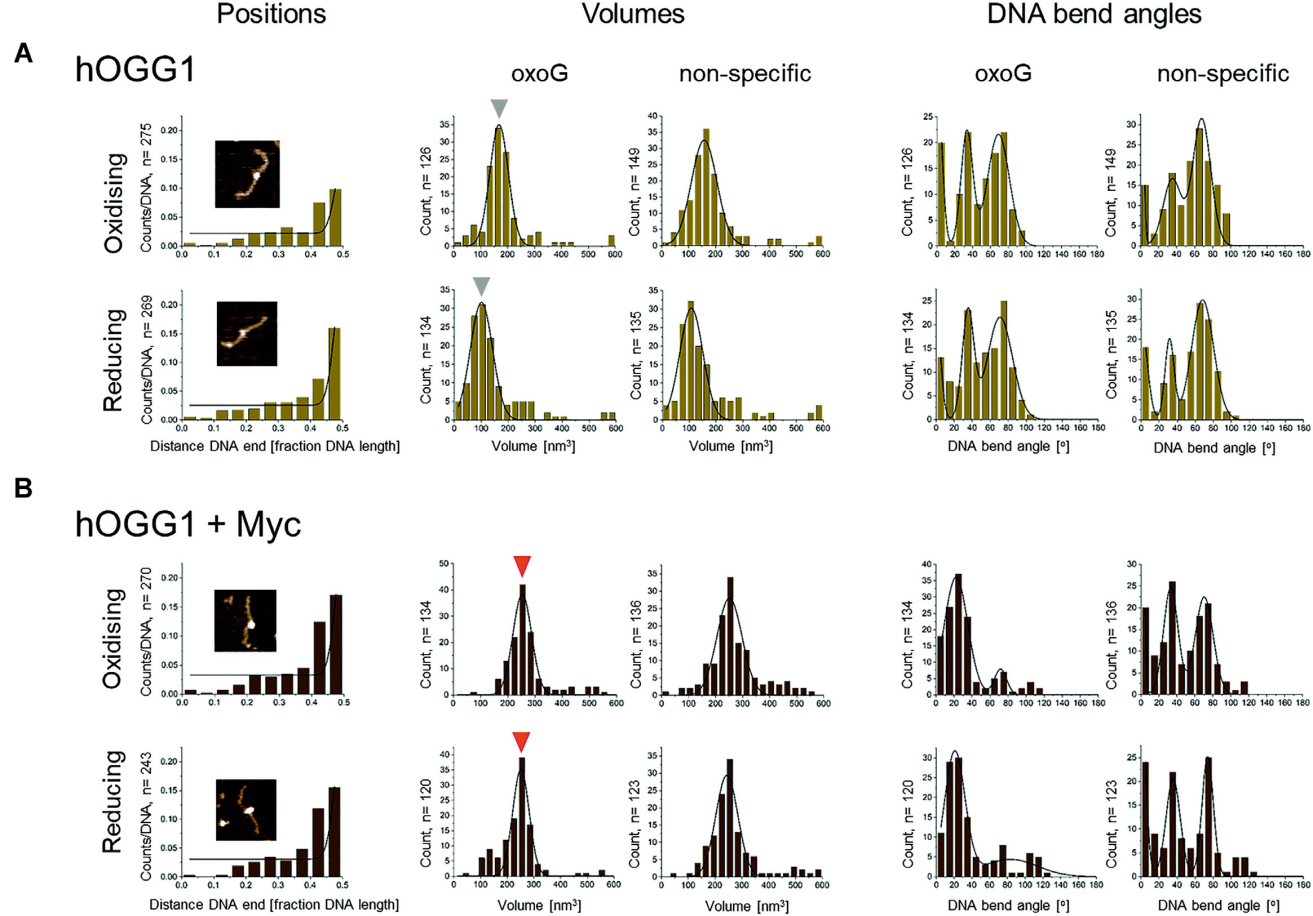
**Myc forms complexes with hOGG1 on DNA and induces conformational changes in hOGG1-oxoG complexes**. Left: Protein binding positions on oxoG DNA (with the oxoG lesion at 50% of the DNA length) from AFM images; middle: AFM volumes of protein complexes on DNA (at the lesion, i.e. at 50% DNA length, and on non-specific DNA); right: DNA bend angles introduced by protein complexes at the oxoG lesion and on non-specific DNA for (**A**) hOGG1 and (**B**) hOGG1 and Myc. Data are pooled from triplicate experiments using 150 nM hOGG1 ± 30 nM Myc. From binding positions (left column), specificities of hOGG1 binding to oxoG (over non-specific DNA) were calculated to be 368 ± 74 under oxidising and 543 ± 37 under reducing conditions (standard deviations from triplicate experiments). These numbers correlate with oxoG occupancies per DNA of 0.10 and 0.18 hOGG1 for oxidising and reducing conditions, respectively (Table 1). Volume analyses (central columns) were consistent with a dimer for pre-oxidised hOGG1 (∼160 nm^3^) and a monomer under reducing conditions (∼100 nm^3^). Volumes of DNA bound complexes in hOGG1 + Myc samples were ∼240 nm^3^ under oxidising as well as reducing conditions. Volume increases correlated with protein sizes (39 kDa for hOGG1 and 76 kDa for GST-tagged Myc). Volumes did not depend on binding to oxoG lesions or non-specific DNA and were identical for wild type and catalytically inactive mutant hOGG1 (Supplementary Figure S9). Consistent with the fact that hOGG1/Myc complexes, once formed, were identical under oxidising and reducing conditions, the AFM position distributions provided identical occupancies of oxoG lesions (0.17/DNA) and similar specificities for oxoG binding, 423 ± 155 for oxidising and 517 ± 161 for reducing conditions. DNA bend angle measurements (right columns) showed DNA bending of ∼0°, ∼35° and ∼70° by both hOGG1 monomer and dimer (under reducing and oxidising conditions, respectively) as well as by hOGG1/Myc complexes at non-specific DNA positions. At oxoG lesions, hOGG1 in the absence of Myc showed comparable DNA bending as for non-specific DNA sites, however, hOGG1/Myc complexes bound at oxoG displayed almost exclusively a single conformational state with DNA bend angle of ∼20^o^. DNA bend angles did not depend on hOGG1 catalytic activity (Supplementary Figure S9) or the absence or presence of Max (Supplementary Figure S11).

Next, we investigated hOGG1 interactions with Myc at oxoG lesions in DNA as well as on undamaged DNA under oxidising and reducing conditions using single molecule AFM imaging. For this, we incubated hOGG1 (150 nM) with or without Myc (30 nM) and DNA containing an oxoG lesion at 50% of its length (Figure 3). Positional analyses (Figure 3A left columns) showed higher specificity of hOGG1 for its target lesion under reducing compared to oxidising conditions (specificities of ∼540 *versus* 370, see Figure 3), consistent with binding affinities from fluorescence polarisation data (Figure 2B).

AFM volume analyses supported monomeric hOGG1 under reducing conditions and dimeric hOGG1 under oxidising conditions (∼100 and 160 nm^3^, respectively), on undamaged DNA as well as at the oxoG lesion (Figure 3A middle columns), consistent with EMSAs (Figure 2C). Consistent with a role of C28 in oxidation-induced hOGG1 dimerisation, the C28A mutant of hOGG1 showed exclusively monomeric complexes on undamaged DNA as well as at the oxoG lesion under oxidising conditions in AFM volume analyses (Supplementary Figure S1E). In addition, these oxidised hOGG1 C28A monomers displayed oxoG binding frequencies that were comparable to hOGG1 wildtype monomers under reducing conditions (Supplementary Figure S1E and Table 1), consistent with binding affinities for oxoG containing DNA substrate from fluorescence polarisation (Supplementary Figure S1B).

**Figure 4.**
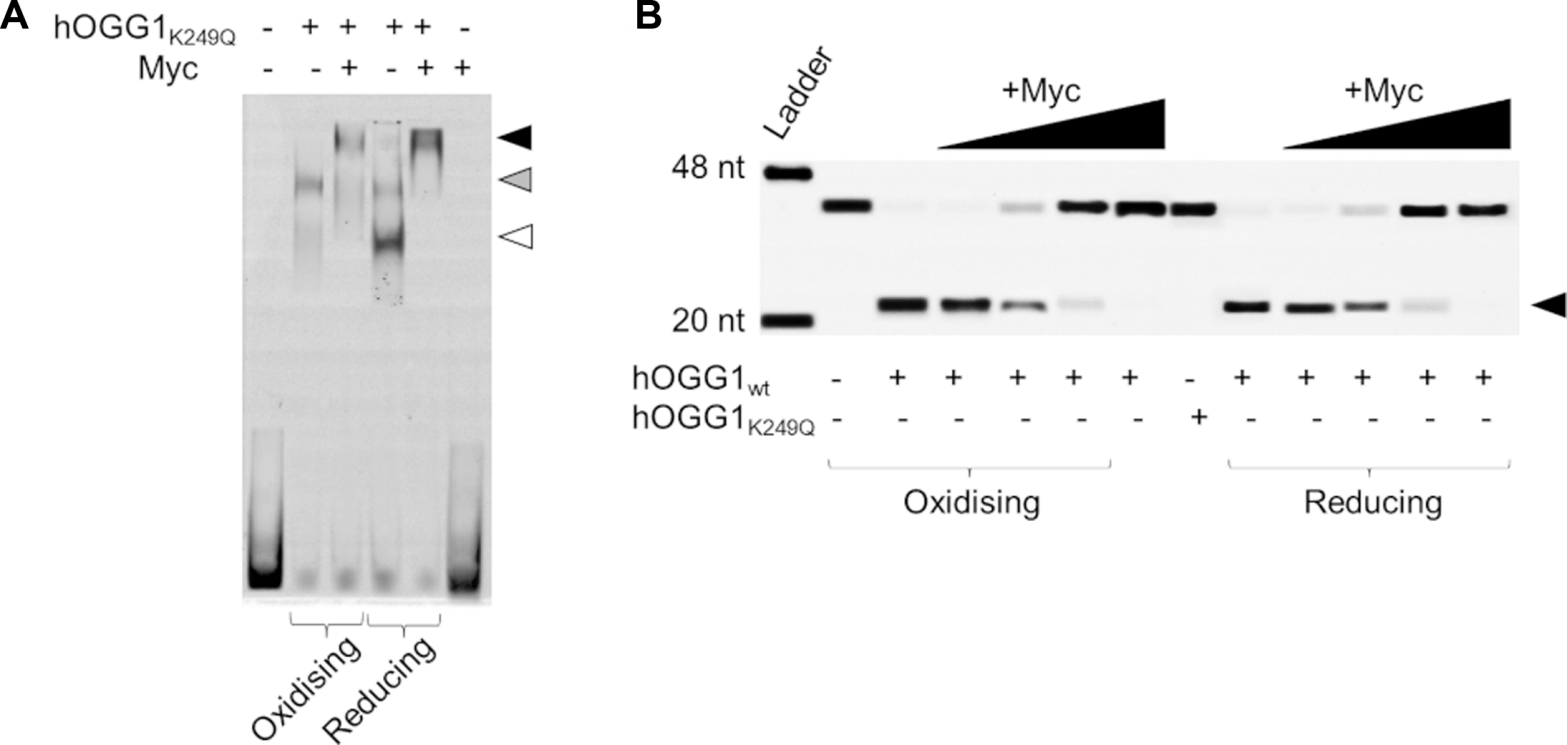
**hOGG1 catalytic activity is suppressed by hOGG1-Myc complex formation on DNA**. (**A**) EMSA indicates comparable sizes of hOGG1/Myc complexes (black arrow) on oxoG containing DNA under oxidising and reducing conditions (see also Supplementary Figure S10). Under oxidising condition (using pre-oxidised hOGG1), hOGG1 forms almost excusively dimers on the DNA (grey arrow) *versus* predominantly monomeric hOGG1 under reducing conditions (+5 mM DTT, white arrow). Protein concentrations: 300 nM hOGG1, 300 nM Myc. (**B**) Titrations of Myc (50–300 nM) to hOGG1 (300 nM) and oxoG DNA (20 nM) samples lead to complete blocking of hOGG1 oxoG repair activity at ∼200 nM Myc. As a control, the catalytically dead hOGG1_K249Q_ variant (300 nM, without Myc, under ambient conditions) is also included (in the gel center). The arrow indicates the repair product.

In the presence of Myc, complex volumes were increased (∼240 nm^3^, Figure 3B middle columns, compare to volumes in Figure 3A), indicating hOGG1-Myc complex formation on DNA. Interestingly, although hOGG1 (wildtype or the catalytically inactive K249Q mutant) alone formed monomeric complexes on DNA under reducing but dimeric complexes under oxidising conditions, for hOGG1/Myc complexes we measured comparable volumes under oxidising and reducing conditions (Figure 3 and Supplementary Figure S9 middle columns). This finding was also supported by identical DNA shifts for hOGG1/Myc complexes under oxidising and reducing conditions in EMSAs (Figure 4A and Supplementary Figure S10). The EMSAs further showed concentration dependent, lower stability of hOGG1/Myc complexes on DNA (both at an oxoG lesion and on undamaged DNA) under reducing conditions (Supplementary Figure S10). In addition, we carried out AFM control experiments with oxoG DNA, hOGG1 and Myc, including Max to allow for DNA interactions by Myc/Max. These data showed comparable oxoG binding frequencies and specificities for hOGG1/Myc/Max complexes as for the hOGG1/Myc complexes above, albeit slightly lower under reducing conditions (Table 1 and Supplementary Figure S11). AFM volumes of DNA bound complexes (both at the oxoG lesion and on undamaged DNA) were increased by the size of Max relative to hOGG1/Myc complexes (Supplementary Figure S11), consistent with complexes of dimeric hOGG1, Myc and Max. Together with the observation that eliminating hOGG1 dimerisation (using the hOGG1 C28A mutant) decreased hOGG1-Myc interactions under oxidative conditions (Supplementary Figures S1D and S7C), these data support the presence of two monomers of hOGG1 in the heteromeric hOGG1/Myc(/Max) complexes, which may serve to stabilise the hOGG1–Myc binding interface.

To characterise conformational properties of hOGG1 and hOGG1/Myc complexes on oxoG lesions and on undamaged DNA, we measured bending introduced into the DNA by the proteins. We and others have previously shown a dynamic equilibrium between three different DNA bend angle states (∼0°, ∼35° and ∼70°) in hOGG1 complexes on undamaged DNA during lesion search (41,53,54). The ∼0° DNA bend angle state (straight DNA) has been interpreted as the hOGG1 lesion search complex conformation (41,53,54). The strongly kinked, ∼70° bend angle state is consistent with the interrogation or excision complex conformation seen in crystal structures of hOGG1 crosslinked to undamaged DNA with the target base flipped into an exo site pocket in hOGG1, or bound to an oxoG lesion with the target base flipped into the catalytic site pocket of hOGG1 (55,56). We interpret the ∼35° DNA bend angle state as an intermediate on the path to formation of the excision competent complex conformation. Extension of AFM analyses to specifically bound hOGG1 at oxoG lesions in our present studies shows the same bend angle states as for undamaged DNA (Figure 3A right columns). These findings are consistent with previous reports for another glycosylase, TDG, which also showed comparable equilibria between bend angle states for undamaged DNA and at the target lesion, and suggest continuous probing for DNA damage by glycosylases during lesion search (54). However, for hOGG1, our AFM analyses demonstrate the stabilisation of the conformation with intermediate (∼35°) DNA bending specifically at oxoG lesions. This result is consistent with a recent crystal structure of hOGG1 bound at an oxoG lesion in DNA (57), in which the DNA is bent but the lesion is in an intrahelical conformation prior to base flipping by hOGG1. Interestingly, Myc binding does not majorly affect DNA bending by hOGG1 at undamaged DNA sites, but strongly affects bending in complexes bound at an oxoG lesion (Figure 3B, right columns). In hOGG1/Myc complexes at the oxoG lesion, the striking, almost complete depletion of the strongly kinked, 70° DNA bend angle state suggests suppression of hOGG1 extrahelical base interrogation and excision (compare DNA bend angles at oxoG in Figure 3A and B). These results suggest that the Myc induced conformational changes in hOGG1 complexes at oxoG lesions may lead to suppression of hOGG1 activity in oxoG lesion repair. Indeed, oxoG repair by hOGG1 was completely inhibited by Myc (as well as by Myc/Max) in activity assays (Figure 4B and Supplementary Figure S12A–C). This suppression of hOGG1 catalytic activity by Myc also persisted in the presence of APE1 (Supplementary Figure S12D), which in BER replaces hOGG1 from its abasic site products after oxoG repair and further processes these abasic sites involving DNA backbone incisions. Complexes of hOGG1/Myc at oxoG lesions adopted a new conformational state, which was characterised by a DNA bend angle of ∼20°, indicating Myc induced conformational changes in the complexes specifically at the target lesion (Figure 3B). Interestingly, the conformational change in hOGG1/Myc complexes did not depend on oxidative or reducing conditions (Figure 3B) or on hOGG1 catalytic activity (Supplementary Figure S9B). We also did not observe any effects of Max on the properties of the complexes (Supplementary Figure S11). However, only the heterodimer of Myc/Max is then able to stably engage with its E-box binding site to initiate transcription (58).

### oxoG-bound hOGG1 enhances Myc-Max recruitment to a downstream E-box motif

**Figure 5.**
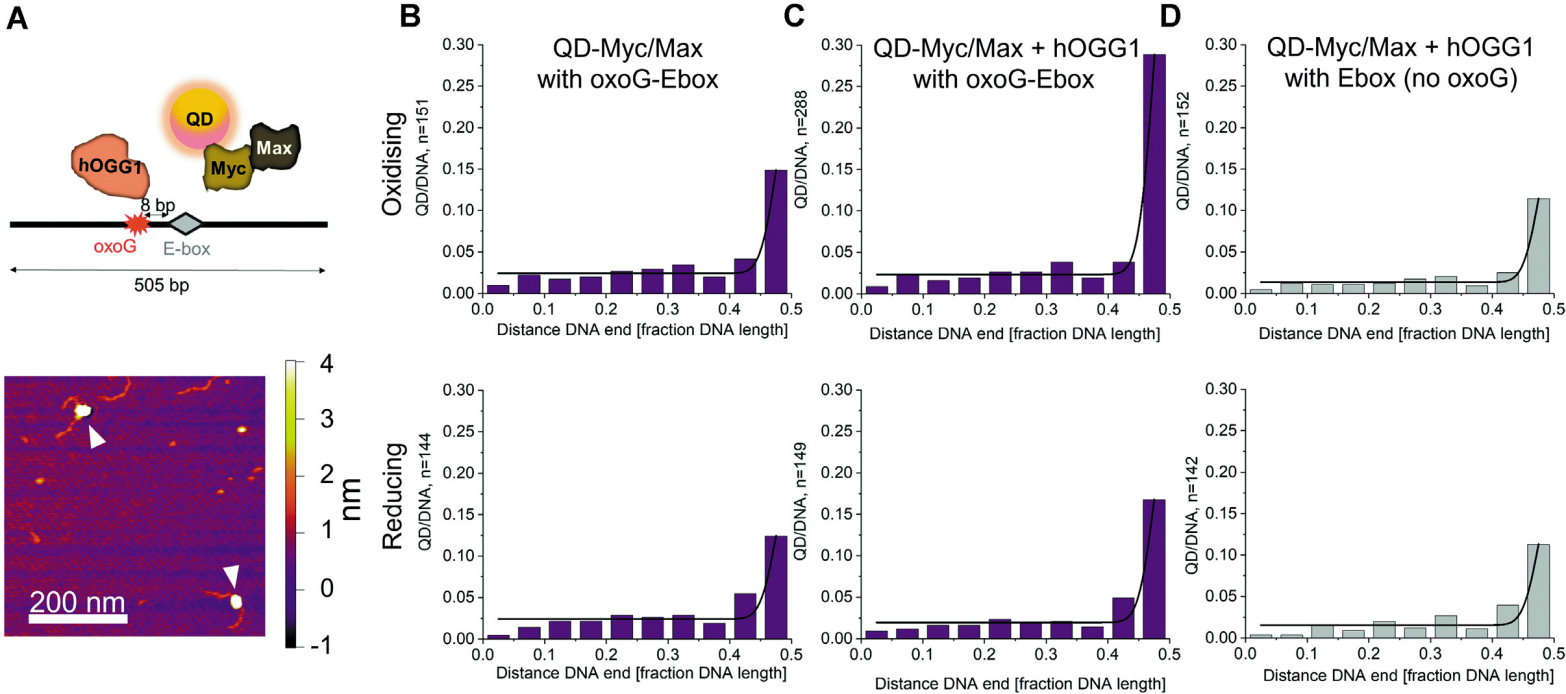
**Myc/Max recruitment to E-box motif is enhanced in the presence of hOGG1 and an upstream oxoG lesion**. (**A**) Experimental setup. Top schematic: Myc is conjugated to a quantum dot (QD) and incubated with Max, hOGG1 and oxoG–E-box (oxoG and E-box spaced by 8 bp) containing DNA substrates. Bottom: An exemplary AFM image depicting QD labeled Myc in complex with DNA (white arrows). The distinct topographical signals of the QDs allow for easy identification of Myc in heterogeneous samples of Myc, Max, and hOGG1 (Supplementary Figure S3A). (**B–D**) Position distributions of QD-Myc on DNA serve to quantify Myc/Max binding at the E-box with an upstream oxoG lesion in the absence (B) or in the presence of hOGG1 (C), and in the presence of hOGG1 but at an E-box without oxoG lesion (D). Data are normalised to the number of DNA molecules analysed (QD/DNA) to directly quantify binding, showing increased Myc/Max loading at E-boxes in the presence of an oxoG lesion and hOGG1 exclusively under oxidative conditions. Both the oxoG and the Ebox are located at ∼50% of the DNA length (see Materials and Methods) and cannot be distinguished in these experiments. However, Myc/Max recruitment frequency to the oxoG-Ebox motif is slightly but significantly higher than the sum of binding to either oxoG (Supplementary Figure S3) or E-box alone (see Table 2 and Supplementary Table S4). Further controls with catalytically inactive hOGG1 K249Q and dimerisation incompetent hOGG1 C28A variants, as well as QD-Myc/Max and hOGG1 with DNA substrate containing exclusively an oxoG lesion (no E-box) and QD/Myc in the absence of Max or hOGG1 are shown in Supplementary Figure S3. Different frequencies for oxoG-E-box, E-box and oxoG binding by Myc/Max (in the absence or presence of hOGG1 wildtype or mutants and under oxidising and reducing conditions) are summarised in Table 2.

To investigate Myc recruitment to its E-box binding motif by hOGG1 we employed quantum dot (QD) labeling of Myc in mixed samples containing Myc as well as its binding partner Max in the absence or presence of hOGG1. We used a DNA substrate that contained an oxoG lesion 8 bp upstream of an E-box motif. The distance between the oxoG lesion and the E-box motif was designed based on previous findings of enhanced binding of transcription factor NFκB to its binding site in DNA in the presence of an oxoG lesion and hOGG1 (18). The QD label on Myc allowed us to unambiguously mark Myc on the DNA substrates by its distinct, high topographical signal using a height cutoff of 3 nm as selection criterion to distinguish QD-Myc(/Max) containing complexes from those containing only hOGG1 in heterogeneous samples (Supplementary Figure S3A). Consistent with previous reports (58), Myc in the absence of Max was not able to stably bind to its E-box binding motif (Supplementary Figure S3B). Max was therefore included in these experiments. Positional analyses revealed significant enhancement of QD-Myc/Max at the oxoG-E-box compared to the E-box without an upstream oxoG lesion by hOGG1 specifically under oxidising conditions (using pre-oxidised hOGG1, Figure 5, Table 2 and Suppl. Table S4). Myc/Max enhancement at the E-box strictly depended on the presence of the oxoG lesion in the DNA substrate (Figure 5, compare C and D) as well as oxidised hOGG1 (Figure 5, compare C and B). In the absence of the E-box motif, Myc/Max was still recruited to the oxoG lesion by hOGG1; however, the amount of Myc/Max recruited to the oxoG-bound hOGG1 was not sufficient to explain the enhancement of oxoG-E-box binding by Myc/Max (Supplementary Figure S3D). Importantly, QD-Myc/Max enhancement at the E-box was not affected by hOGG1 catalytic activity (Supplementary Figure S3E). Furthermore, the C28A mutant of hOGG1 that does not support hOGG1 dimerisation (Figure 1D) did not induce increased Myc binding to the E-box under oxidising conditions (Supplementary Figure S3C and Table 2), again supporting a role of hOGG1 dimerisation in the enhancement of Myc recruitment.

**Table 2.** E-box binding from AFM positional analyses with QD labeled Myc

	Redox condition	Myc/DNA at (oxoG-) E-box	SD	Significance
Myc/Max, oxoG-E-box	Oxidising	0.14	0.002	N/A
	Reducing	0.12	0.01	n.s. (down)
hOGG1-Myc/Max, oxoG-E-box	Oxidising	0.29	0.002	***
	Reducing	0.17	0.008	**
hOGG1-Myc/Max, E-box	Oxidising	0.11	0.01	*** (down)
	Reducing	0.11	0.01	*** (down)
hOGG1-Myc/Max, oxoG	Oxidising	0.15	0.002	*
	Reducing	0.06	0.01	*** (down)
hOGG1-Myc, oxoG-E-box	Oxidising	0.12	0.0002	*** (down)
	Reducing	0.08	0.002	*** (down)
hOGG1_K249Q_-Myc/Max, oxoG-E-box	Oxidising	0.29	0.003	***
	Reducing	0.17	0.01	***
hOGG1_C28A_-Myc/Max, oxoG-E-box	Oxidising	0.16	0.001	***

[QD-Myc] 2 nM, [Max] 2 nM, [hOGG1] 150 nM (where present).

SD standard deviation from triplicate experiments, N/A not applicable.

Significance levels shown relative to Myc/Max and oxoG–E-box substrate at oxidising conditions, n.s. not significant, see Supplementary Table S4 for details.

### The hOGG1-Myc interaction may be responsible for tumourigenic processes

**Figure 6.**
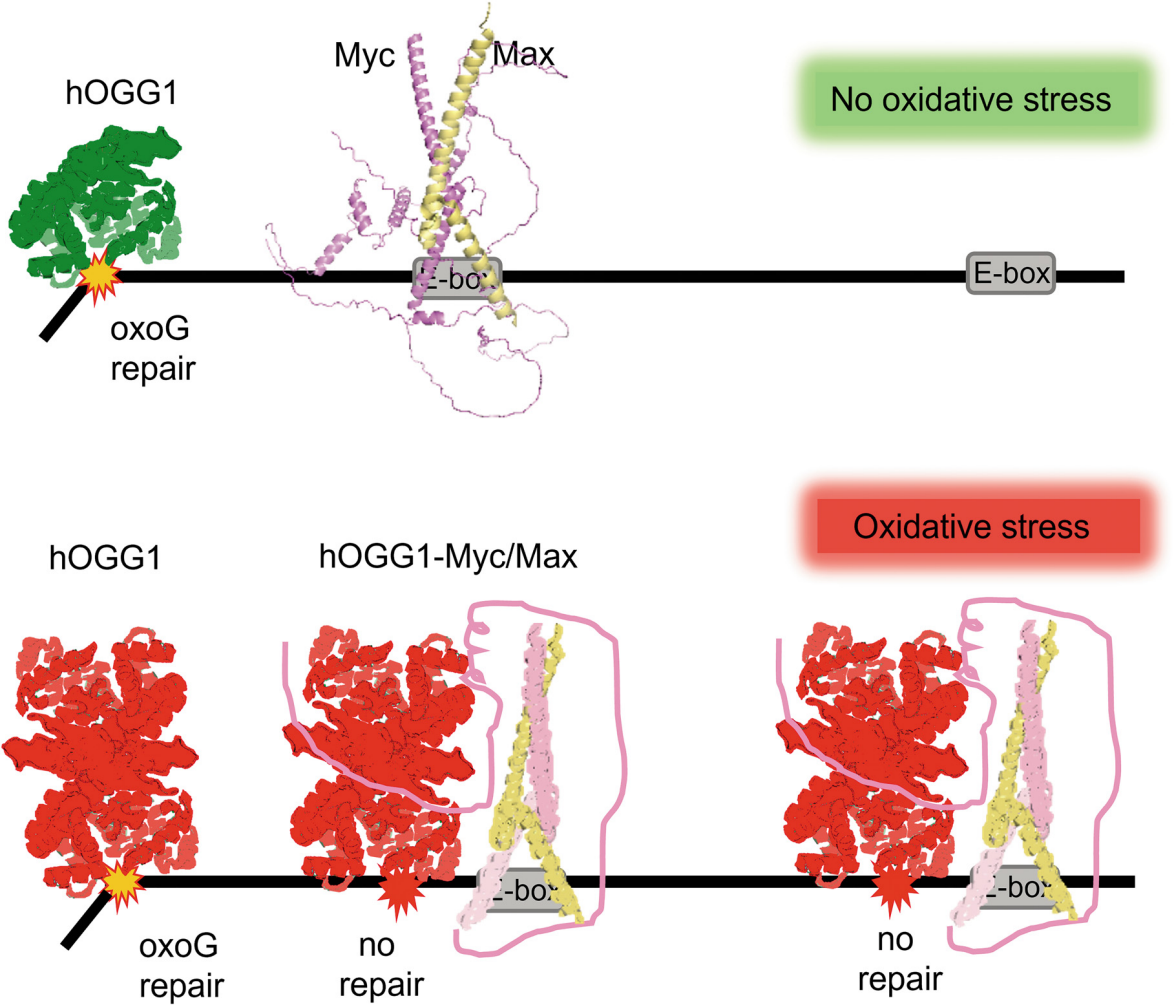
**Model of Myc recruitment by hOGG1**. Oxidative environment introduces oxoG lesions in DNA (red splashes on the DNA) and leads to the dimerisation of hOGG1. Dimers of hOGG1 stabilise interactions with Myc and conformational changes in the complex induced by Myc suppress catalytic oxoG repair activity by hOGG1 (red filled splashes represent unrepaired oxoG). The conformational changes also mediate loading of Myc/Max on the DNA at an oxoG lesion to facilitate Myc/Max binding to its cognate E-box motif present in close vicinity of the lesion. Red and green models indicate oxidised and reduced hOGG1, respectively. The hOGG1 monomer and dimer models are based on the monomer crystal structure (PDB id: 1ko9). The dimer was assembled in pymol (DeLano Scientific; dimerisation at the C28 residue). The Myc-Max complex model (Myc in pink, Max in yellow) in the top (non-oxidising condition) scenario was assembled from the predicted structure of Myc (AlphaFold database) and the crystal structure of the DNA bound complex of the C-terminal DNA binding domain of Myc and N-terminal DNA binding domain of Max (PDB id: 1nkp). The schematically indicated interaction interface between hOGG1 and Myc is based on molecular docking of monomeric hOGG1 and Myc using the Haddock 2.2 webserver (Bonvin Lab) and on the stabilisation of the hOGG1 dimer by the N-terminal part of Myc (Supplementary Figure S5A).

We summarise our findings in a mechanistic model of Myc recruitment to its E-box binding motif in gene promoters by hOGG1 under oxidative conditions (Figure 6). Oxidative stress introduces oxoG lesions in DNA and leads to the dimerisation of hOGG1, which stabilises interactions with Myc. At an oxoG lesion, hOGG1 inactivation by Myc then leads to persistent binding and conformational changes that mediate Myc(/Max) loading on the DNA, for instance at its E-box recognition sequence in promoters in close vicinity downstream of the lesion.

Previous studies have shown that histone demethylation by LSD1, which opens the chromatin at gene promoters, results in the production of ROS that can locally oxidise the DNA (11). The local oxidative environment further leads to the oxidation and dimerisation of hOGG1 (Figure 1). Decreased affinities between oxidised (dimeric) hOGG1 and LSD1 (Figure 1A) may then support dissociation of hOGG1 (or a preformed hOGG1/Myc complex) from LSD1/hOGG1(/Myc) complexes to release the enzyme(s) for binding to the induced oxoG lesions. Consistent with only weak affinity between hOGG1 and LSD1 once hOGG1 is bound to the DNA, AFM experiments required crosslinking of LSD1 and hOGG1 to maintain LSD1/hOGG1 complexes on DNA (Supplementary Figure S13). Myc/Max loaded by hOGG1 at gene promoters can then activate the transcription machinery (59). Consistent with this model, cellular studies have shown co-localisation of hOGG1, Myc, and LSD1 on gene promoters and enhanced gene transcription under oxidative stress that depended on hOGG1 and Myc (6,11,12,16).

In addition to its function as a sequence specific transcription factor (by binding to E-box motifs) for the control of Myc target gene expression, Myc has also been shown to be a global amplifier of transcription from active promoters by amplifying transactivating transcription factors such as estrogen receptors (11,60,61), which stimulate, for instance, the expression of genes involved in the inhibition of cell apoptosis (62). Myc has been shown to recruit LSD1 to chromatin regions with high methylation and acetylation levels of histone 3 (H3) (63). LSD1 then demethylates H3K4 and K9 (11) to initiate the cascade of local, oxoG-induced hOGG1/Myc driven assembly of the transcription machinery and enhancement of Myc target gene expression (12) or, for instance, hormone receptor activated gene expression (11). LSD1 would thus provide spatial control over oxoG induction in DNA directed by Myc to gene promoters. In this context, LSD1 overexpression has been observed in cancer cells and has been linked to enhanced cancer malignancy (64,65). Our model does, however, not strictly depend on LSD1 activity. Under inflammatory conditions, oxidative stress prevails throughout the cell nucleus. Spatial control over oxoG induction in this context is provided by GC rich and G quadruplex forming sequences that are frequently found in the vicinity of gene promoters, since these are particularly prone to oxidation.

Our mechanistic model may also possess general applicability to transcription factor recruitment to their binding sites in gene promoters by hOGG1 under oxidative conditions. For instance, NFκB and SP1 transcription factors have also been shown to directly interact with hOGG1 (10). Intriguingly, these interactions were also enhanced under oxidative conditions, as seen here for hOGG1 and Myc, and it will be interesting to investigate in future studies whether these interactions also directly inactivate hOGG1 catalytic activity similar as reported here for Myc.

In contrast to enhancement of gene expression by Myc-induced inactivation of hOGG1 at oxoG lesions, in the G4 model (see Introduction), oxoG repair activity by hOGG1 is required for G4 formation. Binding of APE1 to the resulting abasic site in the G4 has been shown to result in non-productive APE1 that can recruit transcription factors and RNA polymerase to the G4 (13,30,31). However, G4s have been implicated in the up- or down-regulation of gene transcription, strongly depending on their sequence and their position with respect to the transcription start site and DNA strand (5,13,30,66). Myc-induced inactivation of hOGG1 repair function and subsequent suppression of G4 formation would thus either inhibit or activate gene transcription depending on the position of the oxoG lesion (and thus the potential G4). Moreover, transcription factor recruitment to more distant enhancer sequences has previously been suggested to require oxoG repair by hOGG1 to provide access sites for topoisomerases (11). Topoisomerases function to relax the DNA facilitating DNA looping to support interactions between enhancing factors and RNA polymerase bound at distant regions on the DNA to activate transcription. Intriguingly, however, Myc itself has recently been shown to directly recruit topoisomerases to promoters and to stimulate topoisomerase activity (67), potentially abrogating a need for hOGG1 repair activity in this context. In general, hOGG1-Myc interactions may thus steer gene expression dependent on specific regulatory elements in the DNA (upstream or downstream of gene promoters) or the presence and location of sequences within promoters that are susceptible to oxidation.

Although this has to be confirmed by *in vivo* studies, the hOGG1 induced enhancement of Myc recruitment to its recognition sequences in promoters in the presence of oxoG lesions observed in our studies likely represents carcinogenic events under the enhanced Myc concentrations found in tumour cells. The inflammatory, oxidative conditions in tumour cells that lead to the upregulation of Myc expression may thus promote tumour growth by further stimulating inflammatory cytokine expression in an oxoG and hOGG1 dependent mechanism (68). Our data provide important new information for a better, molecular level understanding of transcription modulation by hOGG1 under oxidative stress conditions that can now be tested in cellular assays. Specifically, our findings predict that individuals harbouring mutations in C28 of hOGG1 may be partially protected against Myc-induced tumourigenic processes. Importantly, our data also suggest the hOGG1–Myc interface as a potential target of inhibitor development for tumour therapy. Inhibitors of hOGG1 catalytic activity that, importantly, interfere with oxoG binding, have recently been reported to prevent the upregulation of inflammatory gene expression in tumours (69,70). However, targeting the hOGG1-Myc interface instead of suppressing hOGG1 oxoG binding and repair activity would possess the advantage of not sacrificing repair of mutagenic oxoG lesions elsewhere in the DNA (by hOGG1 not in complex with transcription factor), which becomes especially important in the context of the enhanced oxidative stress in tumour cells.

## DATA AVAILABILITY

AFM data and original gels are available at OSF https://osf.io/p46n7/.

## Supplementary Material

gkac796_Supplemental_File
