# Supplementary material for: Direct hOGG1-Myc interactions inhibit hOGG1 catalytic activity and recruit Myc to its promoters under oxidative stress
Source: Nucleic Acids Res. 2022 Sep 26;50(18):10385–98. doi: 10.1093/nar/gkac796 (PMC9561264; doi:10.1093/nar/gkac796)
Supplement: gkac796_Supplemental_File [file gkac796_supplemental_file.pdf]

Supplemental data for

Direct hOGG1-Myc interactions inhibit hOGG1 catalytic activity and recruit Myc to its promoters under oxidative stress

Disha M Bangalore & Ingrid Tessmer*

Rudolf Virchow Center, University of Würzburg, Josef Schneider Str. 2, 97080 Würzburg, Germany

Table S1: List of primers used for generation of bacterial expression constructs

Construct	Sequence (5'- 3')
$hOGG1_{wt}$ fw	TTCCAGGGGCCCATGCCTGCCCGCGC
$hOGG1_{wt}$ rv	GGATCCGGTACCTCATTAGTCCTAGCCTTCCGGCCCTTTGG
pETM14 fw $hOGG1_{wt}$	TAGGACTAATGAGGTACCGGATCCGAA
pETM14 rv $hOGG1_{wt}$	CATGGGCCCCTGGAACAGAAC
$hOGG1_{K249Q}$ fw	TGGGCACCCAAGTGGCTGAC
$hOGG1_{K249Q}$ rv	AGTCAGCCACTTGGGTGCCCA
$hOGG1_{C28A}$ fw	CATCCCGGCTCCTCGCTCT
$hOGG1_{C28A}$ rv	GAGCGAGGAGCCGGGATG
$hOGG1_{C241A}$ fw	AGGCCCTCGCTATCCTGCC
$hOGG1_{C241A}$ rv	GGCAGGATAGCGAGGGCCT
$hOGG1_{C253A}$ fw	AGGTGGCTGACGCTATCTGCCT
$hOGG1_{C253A}$ rv	AGGCAGATAGCGTCAGCCACCT
Myc_{1-163} fw	CAGGGCGCCCCGTATTTCTACTGC
Myc_{1-163} rv	GGATCCGGTACCTCATTAGTCCTAGGCGCTCAGATCCTGCA
pETM41 fw Myc_{1-163}	TAGGACTAATGAGGTACCGGATCCGA
pETM41 rv Myc_{1-163}	CGGGGCGCCCTGAAAATAAAGATTCTCGC

Abbreviations: forward primer (fw), reverse primer (rv)

Table S2: List of oligonucleotides for DNA lesion substrates.

oxoG (top) for EMSA, fluorescence polarisation, and AFM substrate preparation	5‘-GCATGCCTC(oxoG)AGTCTAGAGGTACCAGATCTGATCCTCTA<br>GAGTCGACC-3‘
E-box (top) for AFM substrate preparation	5‘-GCATGCCTCGAGTCTAGAGGCACGTGATCTGATCCTCTA<br>GAGTGCACC-3‘
oxoG E-box (top) for AFM substrate preparation	5‘-GCATGCCTC(oxoG)AGTCTAGAGGCACGTGATCTGATCCTCTA<br>GAGTGCACC-3‘
oxoG for activity assays (top)	5‘-GCATGCCTCGAGTCTAGAGGTACCA(oxoG)ATCTGATCCTCTA<br>GAGTCGACC-3‘
bottom strand for oxoG substrates	5‘-GGTCGACTCTAGAGGATCAGATCTGGTACCTCTAGACTC<br>GAGGCATGC-3‘
bottom strand for E-box and oxoG-E-box substrates	5‘-<br>GGTCGACTCTAGAGGATCAGATCACGTGCCTCTAGACTCGAGGCATGC-3‘

Abbreviations: 8-oxo-guanine (oxoG), enhancer box (E-box)

The E-box sequence motif is underlined. All oligonucleotides are 48 nt long. For EMSA and fluorescence polarisation studies with oxoG DNA substrate, a 5‘ fluorescently labeled bottom strand was employed (Alexa Fluor 647 (AF647) for polarisation, Cy3 for EMSAs). For hOGG1 activity assays, a Cy3 fluorescence label was attached to the 5‘ end of the top (oxoG containing) strand.

Table S3: List of primers used for generation of E-box containing DNA substrates for AFM

E-box fw	GTCTAGAGGCACGTGATCTGATCC
E-box rv	GGATCAGATCACGTGCCTCTAGAC

Abbreviations: forward primer (fw), reverse primer (rv), enhancer box (E-box)

Table S4: t values for student t tests.

	t values	P values
**hOGG1 – protein interactions, oxidising *versus* reducing conditions (polarisation)**		
hOGG1-Myc interactions	6.19	0.0034
hOGG1-LSD1 interactions	3.37	0.0280
hOGG1$_{C28A}$-Myc interactions	0.13	0.9028
hOGG1$_{C241A}$-Myc interactions	3.54	0.0240
hOGG1$_{C253A}$-Myc interactions	5.70	0.0046
**hOGG1 – DNA interactions, oxidising *versus* reducing conditions (polarisation)**		
hOGG1 oxoG binding	12.67	0.0002
hOGG1 non-specific DNA binding	0.09	0.9326
hOGG1$_{K249Q}$ oxoG binding	16.14	0.0001
hOGG1$_{C28A}$ oxoG binding	0.51	0.6369
hOGG1$_{C241A}$ oxoG binding	1.64	0.1764
hOGG1$_{C253A}$ oxoG binding	0.13	0.9028
**Myc (±Max, ±hOGG1) binding to target *versus* Myc/Max binding to oxoG-E-box in the absence of hOGG1 under oxidising condition (AFM)**		
Myc/Max – oxoG-E-box reducing	-2.551	-0.0632
hOGG1/Myc/Max - oxoG-E-box oxidising	85.11	0.0000001
reducing	5.00	0.0075
hOGG1$_{K249Q}$/Myc/Max - oxoG-E-box oxidising	66.61	0.0000003
reducing	6.87	0.0023
hOGG1$_{C28A}$/Myc/Max - oxoG-E-box oxidising	13.21	0.0002
hOGG1/Myc - oxoG-E-box oxidising	-19.35	-0.00004
reducing	-38.26	-0.000003
hOGG1/Myc/Max - E-box oxidising	-8.04	-0.0013
reducing	-5.83	-0.0043
hOGG1/Myc/Max - oxoG oxidising	2.98	0.0406
reducing	-13.99	-0.0002
**Myc/Max-hOGG1 binding events to oxoG-E-box *versus* the sum of binding to oxoG and to E-box (AFM)**		
oxidising	7.16	0.0020
reducing	0.411	0.7020

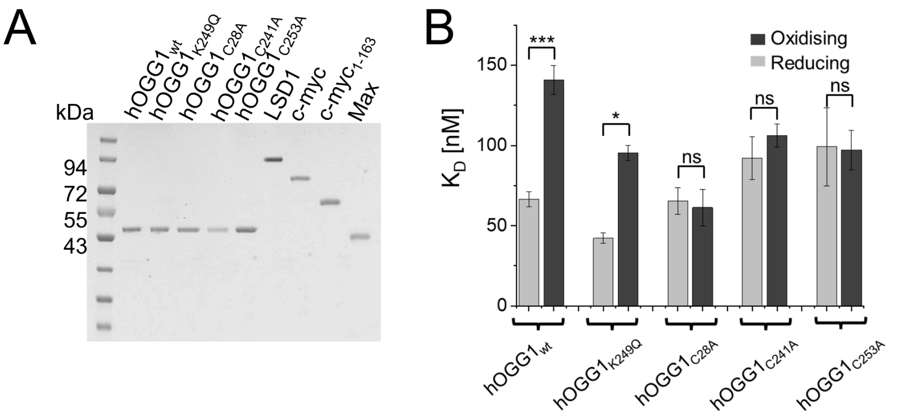

	K_D for oxoG binding [nM]	SD
$hOGG1_{C28A}$ oxidising	61	11
$hOGG1_{C28A}$ reducing	65	8
$hOGG1_{C241A}$ oxidising	106	7
$hOGG1_{C241A}$ reducing	92	13
$hOGG1_{C253A}$ oxidising	97	12
$hOGG1_{C253A}$ reducing	99	24

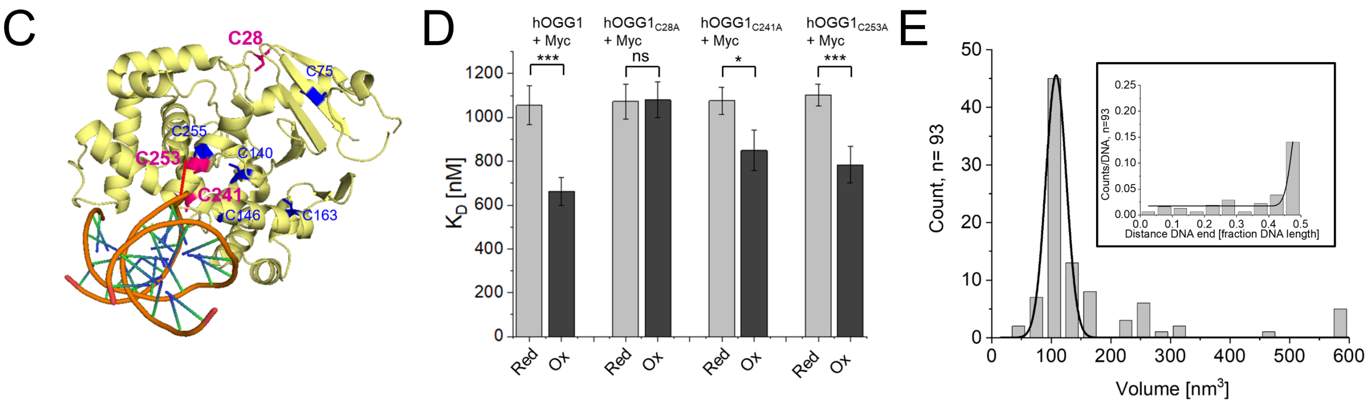

Fig. S1: Purity of all recombinant proteins and functionality of hOGG1 mutants. (A) SDS PAGE gel of all protein variants used. **(B)** OxoG binding of hOGG1 variants under reducing (grey) and oxidising (black) conditions compared to wild type (WT) binding affinity. The K249Q mutant shows enhanced binding likely due to decreased dissociation for this catalytically inactive variant. Under reducing conditions, the C28A mutant shows comparable binding to oxoG as wild type hOGG1, while the other two cysteine mutants (C241A and C253A) display reduced binding to oxoG lesions. C241 and C253 are located in or near the DNA binding interface of hOGG1 as well as close to the catalytic site (C) and their mutation may thus interfere with correct DNA and oxoG interactions. All three cysteine to alanine mutations (C28A, C241A, and C253A) led to complete abrogation of reduced oxoG binding upon hOGG1 oxidation. Data are averages (and standard deviations) from triplicate experiments. On the right, K_D's for oxoG binding by the three cysteine variants are summarised in a table. **(C)** Crystal structure of hOGG1 bound to oxoG in DNA (pdb 6W0M). Accessible cysteines in hOGG1 are highlighted in pink, inaccessible cysteines in blue. **(D)** Interactions of cysteine variants of hOGG1 with c-Myc under reducing (grey) and oxidising (black) conditions. Interactions for wildtype hOGG1 are included as reference. For details on significances see Table S4. **(E)** AFM volume analyses of pre-oxidised hOGG1 C28A showed exclusively monomers on undamaged DNA as well as at an oxoG lesion in DNA. The position distribution (inset) demonstrates high binding specificity (S = 779 ± 471) of this protein variant to oxoG lesions, albeit with lower binding frequency compared to the wildtype enzyme (Fig. 3 and Table 1 in main manuscript).

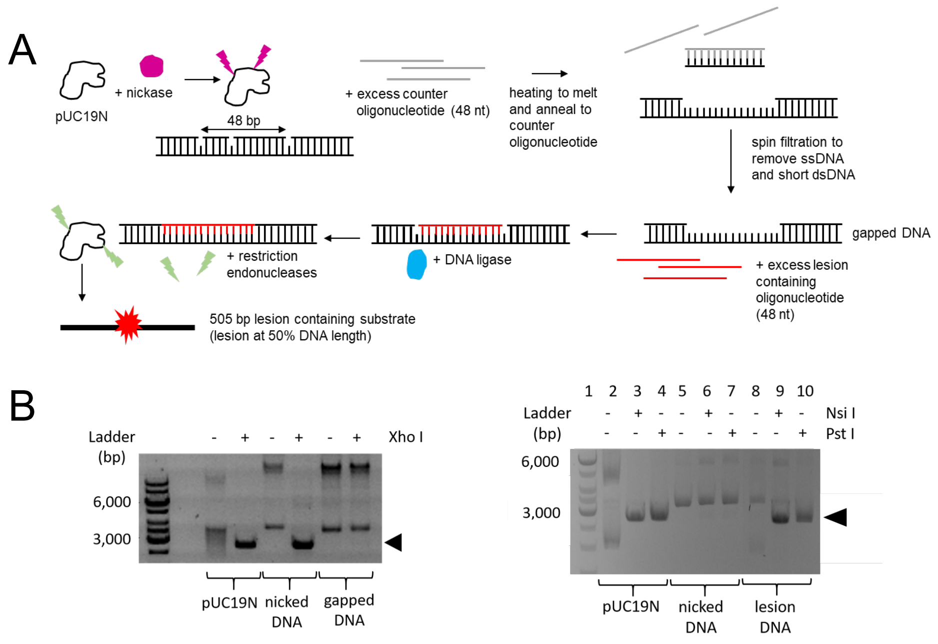

Fig. S2: DNA substrate preparation. (A) Schematic of DNA substrate preparation procedure for AFM. Enzymes used: nickase Nt.BstNBI; T4 DNA ligase; restriction enzymes NdeI and BsaXI. Three closely spaced Nt.BstNBI restriction sites were incised by incubation with the nickase. The single stranded DNA (ssDNA) stretch between the incisions was then removed *via* repeated thermal melting in the presence of excess counter oligonucleotide (bottom strand for oxoG substrates, Suppl. Table S2) and centrifugation through 100 kDa cut-off spin filters (Merck Millipore) in a gapping step. The 48 nucleotide (nt) ssDNA gap was replaced by a modified oligonucleotide, Suppl. Table S2) by incubation at 45°C for 2 h in the presence of a 20-fold excess of the modified oligonucleotide, and ligated with the plasmid overnight with T4 DNA ligase (NEB) at ambient temperature. The ligated product was treated with restriction enzymes NdeI and BsaXI (NEB) resulting in a 505 bp long DNA substrate containing the feature of interest (oxoG, E-box, or oxoG E-box, see also below) at ~50% total DNA length (oxoG at 49.8% if present, E-box at 51.8% if present). The digested products were run on a 1% agarose gel to separate the resulting 505 bp and 2,224 bp digestion products, followed by gel extraction and purification of the 505 bp substrate *via* a commercial kit (Macherey-Nagel). Concentrations were measured using a nanodrop spectrophotometer using an extinction coefficient of 6,700 $M^{-1}cm^{-1}bp^{-1}$. To prepare the DNA substrate containing an E-box motif (CACGTG) or an oxoG lesion as well as the E-box motif placed 8 nt apart, the E-box sequence was cloned into pUC19N plasmid (for primers see Suppl. Table S3) and successful insertion was confirmed by sequencing. The oxoG E-box and E-box substrates were then prepared as described above for oxoG substrate (using the bottom strand for oxoG and oxoG-E-box substrates as counter oligonucleotide in the gapping step). **(B)** All steps were controlled by restriction digestion assays. Left: XhoI restriction enzyme digestion of the DNA substrate at different stages of the preparation confirms complete removal of the 48 nt stretch between Nt.BstNBI nick sites. XhoI can only incise at dsDNA positions and the XhoI restriction site is located in the 48 nt ssDNA region for gapped DNA. Right: Restriction digestion with NsiI and PstI with restriction sites at the 5‘ and 3‘ nicks between original DNA and insert, respectively, confirm complete ligation of the substrate after insertion of the lesion containing oligonucleotide. Original pUC19N and nicked pUC19N serve as positive and negative controls. Arrows indicate successful incisions by XhoI, NsiI, or PstI.

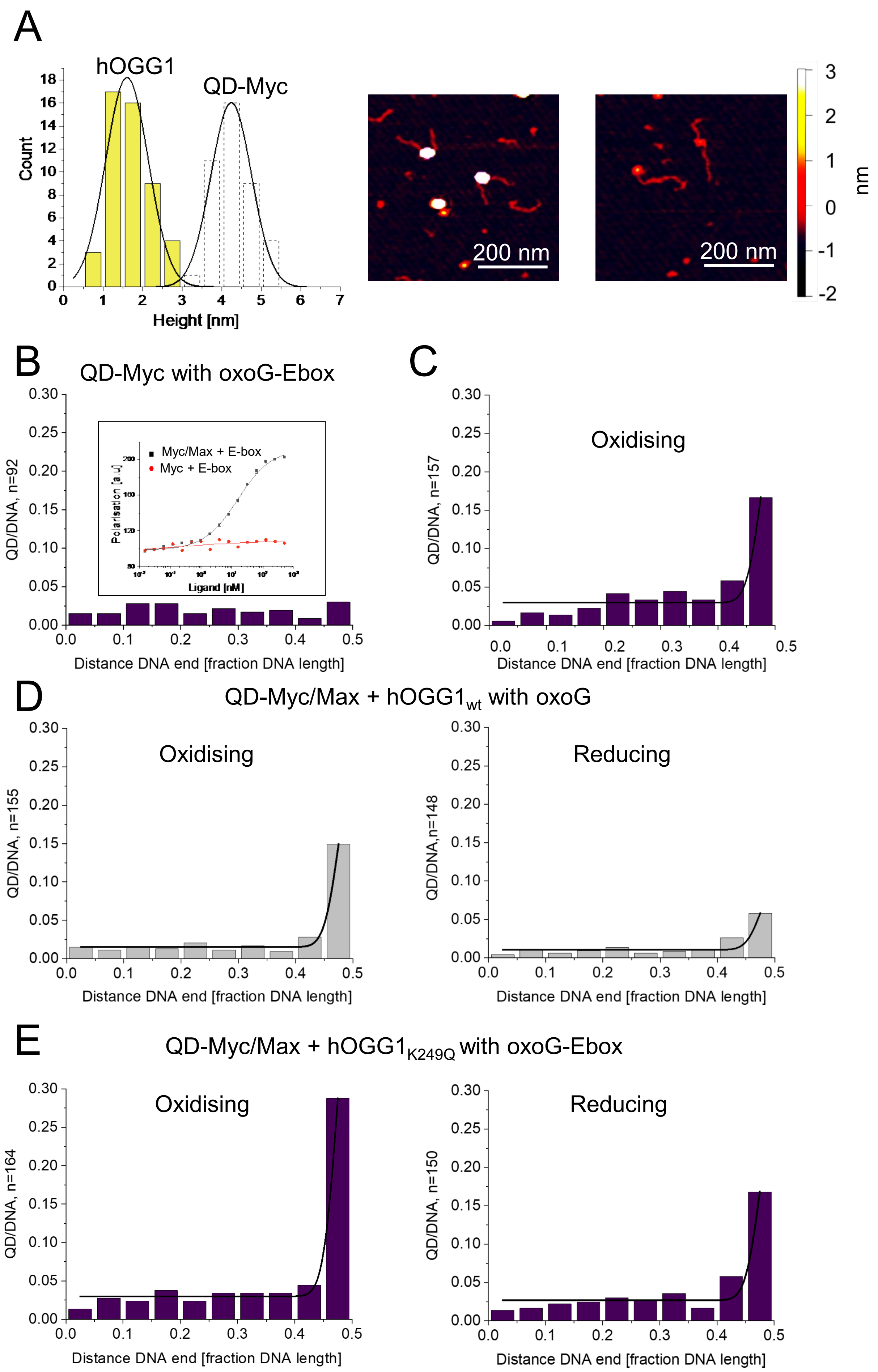
A
hOGG1
QD-Myc
Count
Height [nm]
200 nm
200 nm
nm
B
QD-Myc with oxoG-Ebox
QD/DNA, n=92
Myc/Max + E-box
Myc + E-box
Polarisation [a.u]
Ligand [nM]
Distance DNA end [fraction DNA length]
C
Oxidising
QD/DNA, n=157
Distance DNA end [fraction DNA length]
D
QD-Myc/Max + hOGG1wt with oxoG
Oxidising
QD/DNA, n=155
Distance DNA end [fraction DNA length]
Reducing
QD/DNA,n=148
Distance DNA end [fraction DNA length]
E
QD-Myc/Max + hOGG1K249Q with oxoG-Ebox
Oxidising
QD/DNA, n=164
Distance DNA end [fraction DNA length]
Reducing
QD/DNA, n=150
Distance DNA end [fraction DNA length]

Fig. S3: AFM analyses of QD-Myc –DNA samples. (A) Selection criterion for QD identification in heterogeneous QD-Myc/Max ± hOGG1 samples. QD-Myc bound to DNA showed heights of ~4.5 nm while DNA bound hOGG1 displayed heights around 1.5 nm in topographical AFM images (left). Heights were measured either in samples containing exclusively DNA and QD-Myc (QD-Myc heights) or exclusively DNA and hOGG1 (hOGG1 heights) using the MFP AFM software. Example AFM images are shown for QD-Myc (middle) and hOGG1 (right) incubations with oxoG – E-box DNA (height scale bar on the right). Max has a size similar to hOGG1 so that non-labeled Max can be expected to possess similar heights as hOGG1 in the images. Protein complexes with heights ≥ 3 nm were thus considered to contain QD-Myc. **(B)** Position distribution of QD-Myc (without Max) on oxoG-E-box DNA (with oxoG-Ebox located at ~50% of the DNA length) shows no recognition of the E-box by Myc in the absence of Max. Lack of stable binding to the E-box recognition motif by Myc in the absence of Max is also confirmed by fluorescence polarisation assay (inset). **(C)** Position distribution of QD-Myc/Max on oxoG-E-box DNA in the presence of pre-oxidised hOGG1 C28A. **(D)** Position distributions on oxoG DNA (without E-box) of QD-Myc/Max complexes in the presence of hOGG1 under oxidising (left) and reducing conditions (right). **(E)** Position distributions on oxoG-E-box DNA of QD-Myc/Max complexes in the presence of catalytically inactive hOGG1 K249Q under oxidising (left) and reducing conditions (right) are comparable to those obtained with wildtype hOGG1, demonstrating no effect of hOGG1 catalytic activity on Myc recruitment. For significances of enhancement for Myc/Max binding to the E-box recognition sequence under the different conditions see Table 2 and Suppl. Table S4.

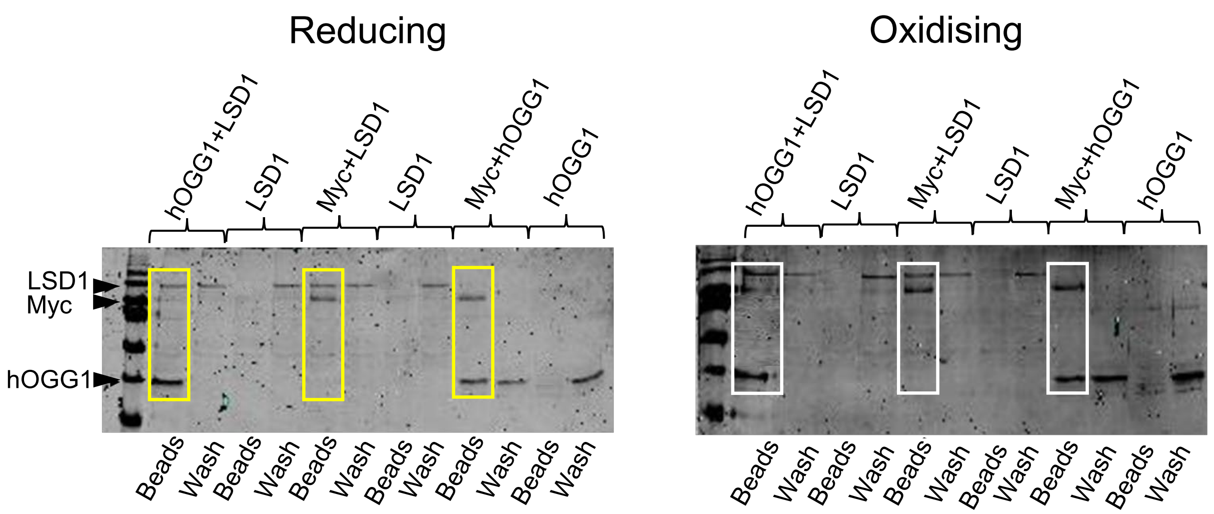

Fig. S4: *In vitro* pull-down assays of hOGG1, c-Myc, and LSD1 interactions. To visualise direct protein-protein interactions between purified proteins, 20 µl of beads (Protino® Ni^{2+}IDA beads for hOGG1-LSD1 interactions and glutatione sepharose beads for Myc-LSD1 and Myc-hOGG1 interactions) were washed thoroughly with incubation buffer. 1.3 µM of the bait protein (his_6 tagged hOGG1 or GST tagged c-Myc) was incubated with the respective beads for 5 min before addition of a ~30-fold excess of prey protein (40 µM of c-Myc, LSD1, or hOGG1) and further incubation for 20 min at room temperature. The his_6 tag was removed from LSD1 prior to pull down by his_6-hOGG1 or GST-cMyc, and from hOGG1 prior to pull down by GST-cMyc *via* 3C protease cleavage. As controls, bait protein-free beads were incubated with prey protein. Sample and control incubations were subjected to 3 washes. Samples (beads and washes) were denatured for 5 min at 95°C and run on a 15% SDS gel. Coomassie stained bands were visualised using a camera based imaging system (Fusion FX6, Vilber). Yellow and white boxes indicate the pulled down samples for reducing and oxidising conditions, respectively. Experiments were performed in triplicate at oxidising and reducing conditions.

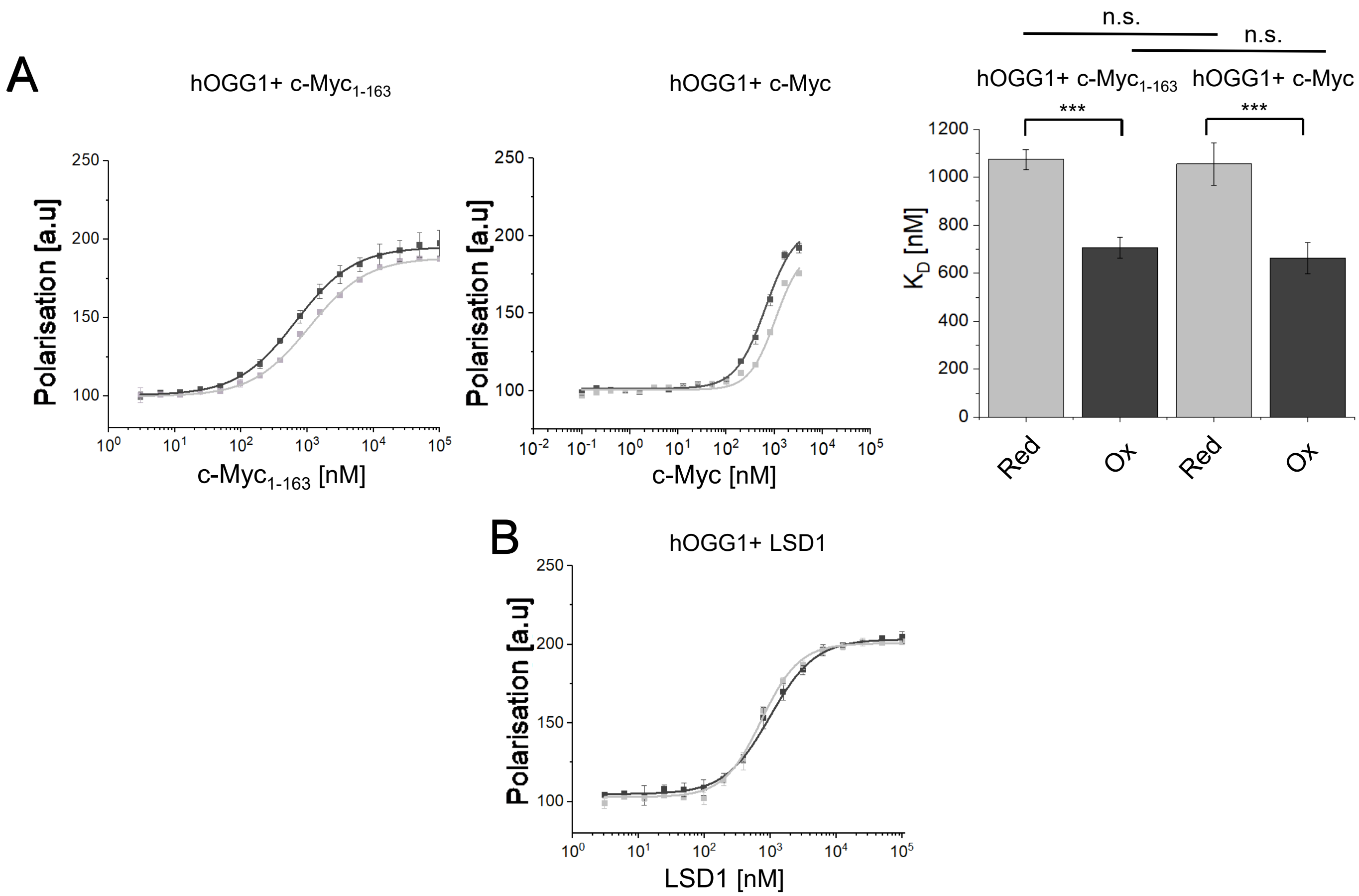

Fig. S5: Polarisation assay of hOGG1 interactions with full length GST tagged c-Myc versus the his$_6$ tagged N-terminal domains of c-Myc, c-Myc$_{1-163}$ and with LSD1. Grey and black indicate reducing and oxidising conditions, respectively. **(A)** Full length c-Myc (centre) and the shortened N-terminal (aa 1-163, left) protein showed comparable binding affinities for hOGG1. The quantification on the right shows significant differences between oxidising and reducing conditions, but no significant differences (n.s.) between full length Myc and Myc$_{1-163}$ (K_D's of 705 ± 43 nM (oxidising) and 1074 ± 41 nM (reducing) for c-Myc$_{1-163}$ *versus* 662 ± 65 nM (oxidising) and 1055 ± 89 nM (reducing) for full length c-Myc). Purified GST tagged full length c-Myc was purchased from Antibodies.com. The gene for c-Myc$_{1-163}$ was cloned from pGEX4T1-Myc$_{1-163}$ plasmid (provided by Elmar Wolf, University of Würzburg) into pETM41 vector, coding for an N-terminal maltose binding protein (MBP) and his$_6$ tag. MBP- his$_6$ tagged Myc$_{1-163}$ was purified by Ni^{2+}-NTA affinity chromatography followed by anion exchange chromatography (MonoQ 10/100 GL) and size exclusion chromatography (Superdex 200 16/600 GL), and eluted in 20 mM Tris-HCl, pH 8.0, 200 mM NaCl, 5% glycerol, 0.5 mM DTT. The protein was purified to > 95% homogeneity as judged from Coomassie stained SDS-PAGE (Fig. S1A) and stored at -80° until use. Concentrations of purified MBP-his$_6$ tagged Myc$_{1-163}$ were determined spectrophotometrically using an extinction coefficient of 87,800 $M^{-1}cm^{-1}$. The MBP-his$_6$ tag was removed from the protein *via* TEV cleavage for fluorescence polarisation assays. **(B)** LSD1-hOGG1 interaction. Data shown in (A) and (B) are averages (with standard deviations) from triplicate measurements and the K_D's plotted in (A) are averages (± standard deviation) from the three individual fits.

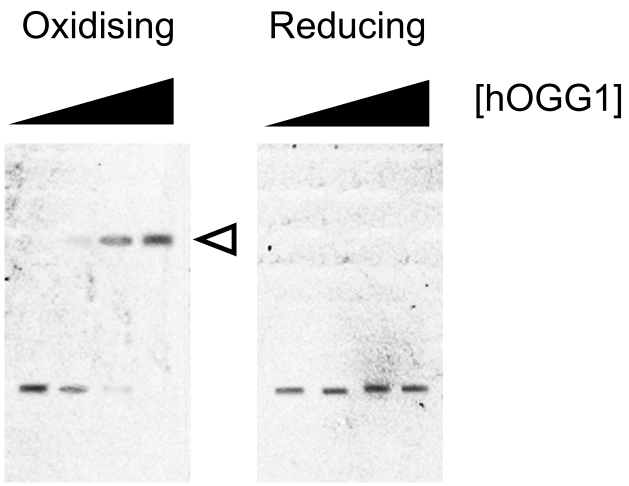

Fig. S6: Dimerisation of hOGG1 under oxidising conditions visualised by non-reducing SDS-PAGE. Alexa Fluor 488 (AF488) fluorescently labeled, pre-oxidised or reduced hOGG1 at 5 nM was titrated with increasing concentrations (100 nM to 750 nM) of pre-oxidized or reduced hOGG1, respectively. The arrow indicates hOGG1 dimer.

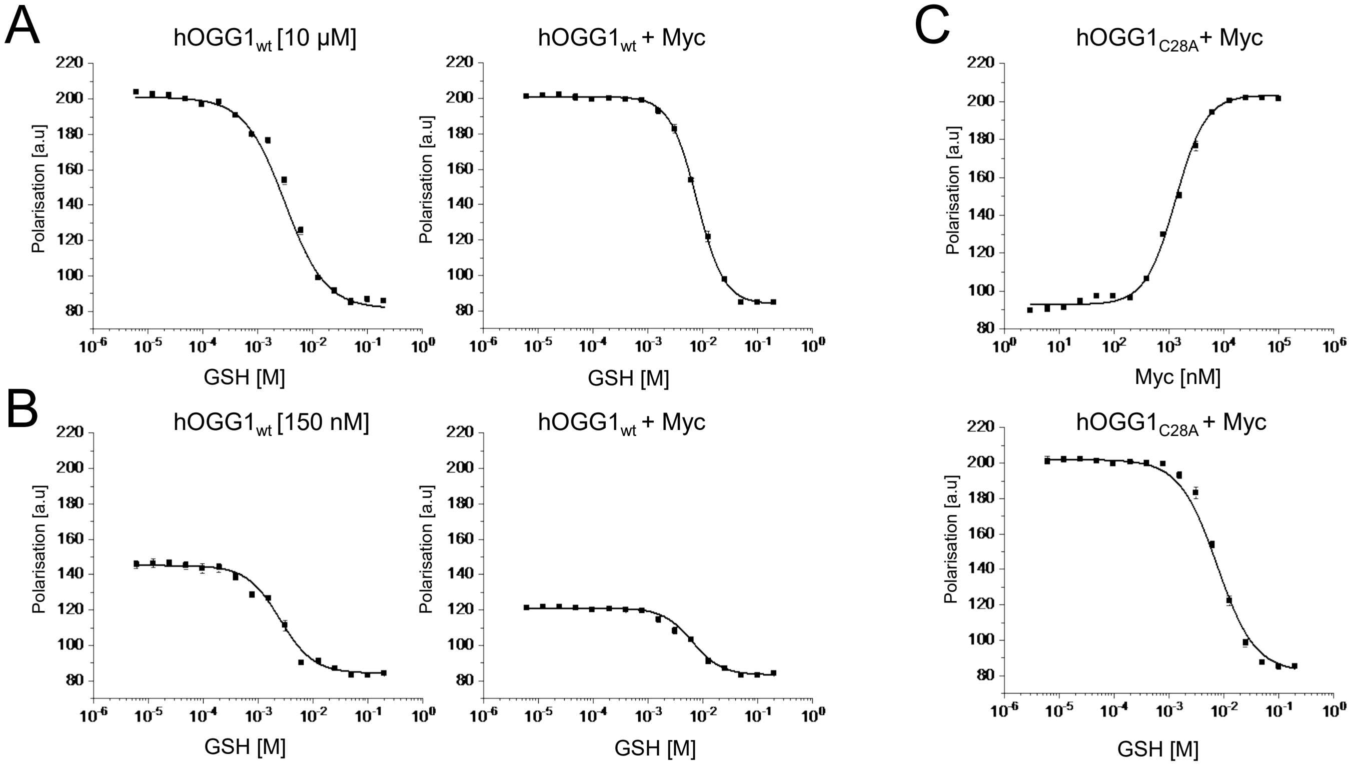

Fig. S7: Stability of hOGG1 dimers and hOGG1/Myc complexes against reduction by glutathione. (A) Fluorescence polarisation measurements showed that glutathione (GSH) only weakly dissociates the hOGG1 dimer (EC50 3.2 ± 0.4 mM, left). Furthermore, the hOGG1-Myc complex (K_D ~ 660 nM under oxidising conditions) that likely contains two monomers of hOGG1 (see main text) was significantly further stabilised against GSH (EC50 7.8 ± 0.2 mM, right). The concentrations of hOGG1 (left) and hOGG1 as well as Myc (right) were 10 µM in these GSH titration. Pre-oxidised hOGG1 was used in these experiments. **(B)** Comparable results were obtained with hOGG1 concentrations of 150 nM (left) and 150 nM hOGG1 as well as Myc in GSH titrations (right). EC50 values for GSH induced complex dissociation were 2.5 ± 0.3 mM and 6.4 ± 0.3 mM for hOGG1 and hOGG1+Myc, respectively, at these lower protein concentrations. **(C)** The C28A mutant of hOGG1 did not support dimerisation in the absence of Myc and formed complexes with Myc with a K_D of ~1.3 µM (top). This affinity is approximately 2-fold weaker than for $hOGG1_{wt}$ under oxidising condition and comparable to $hOGG1_{wt}$ monomer under reducing condition (Fig. 1A). Although hOGG1 C28A in the absence of Myc did not dimerise, the complex of hOGG1 C28A with Myc likely contains two monomers of hOGG1 (see main text) leading to stabilisation of the $hOGG1_{C28A}$–Myc complex under conditions of GSH titrations (EC 7.6 ± 1.3 mM, bottom). For Myc titrations with $hOGG1_{C28A}$, and GSH titrations with $hOGG1_{C28A}$ + Myc, protein concentrations were 10 µM to ensure initial complex formation. Pre-oxidised hOGG1 C28A was used in these experiments. The c-$Myc_{1\text{-}163}$ N-terminal construct of Myc was used in all these titrations.

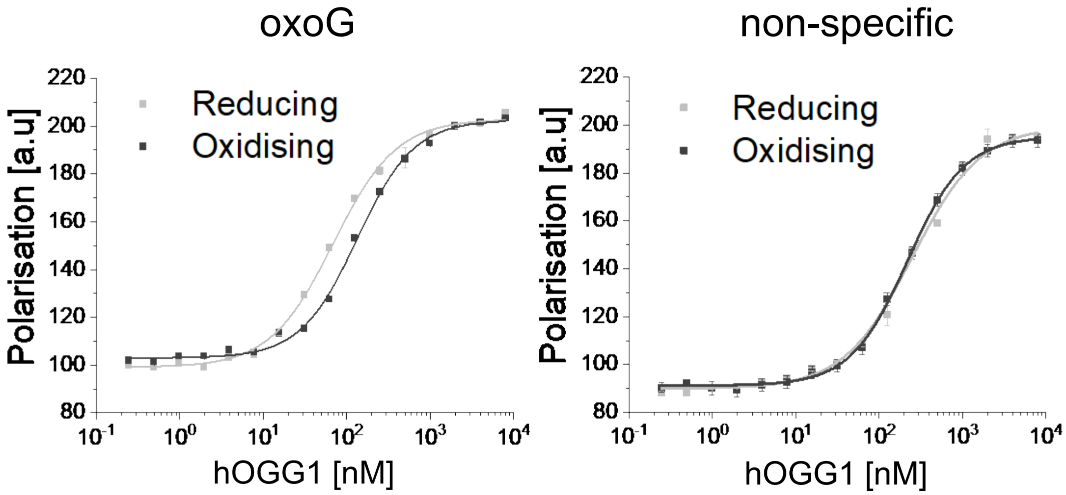

Fig. S8: Fluorescence polarisation data for hOGG1 DNA binding. Left: binding of hOGG1 to oxoG containing DNA, right: hOGG1 binding to non-specific DNA substrate. Curves represent fits to averages from triplicate measurements under oxidising (black) and reducing conditions (grey).

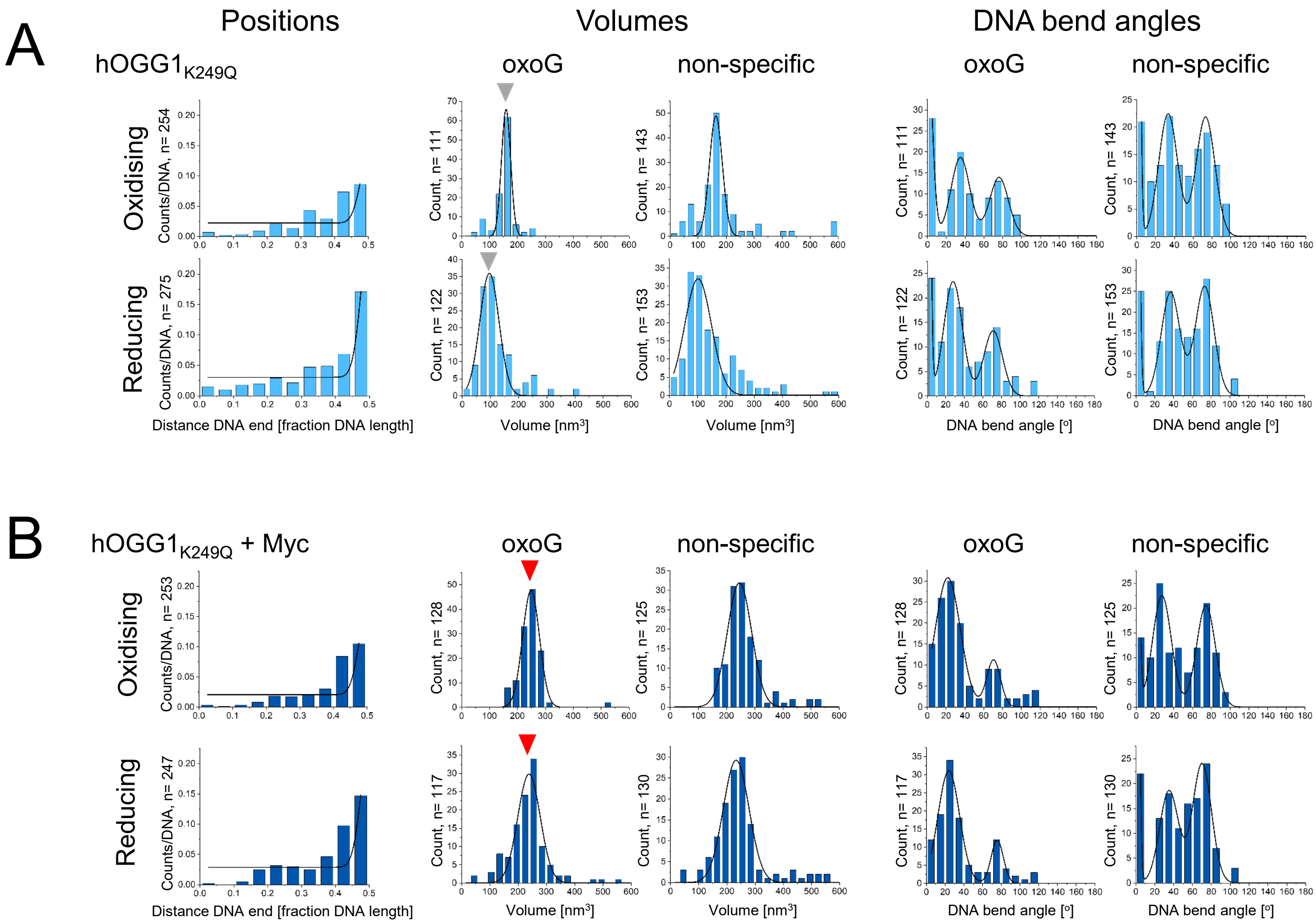

Fig. S9: AFM analyses of inactive hOGG1 K249Q ± Myc on oxoG-DNA. **(A)** hOGG1$_{K249Q}$ on oxoG-DNA. **(B)** hOGG1$_{K249Q}$ and Myc on oxoG-DNA. Pooled data from duplicate experiments under oxidising conditions are shown at the top, and at the bottom for reducing conditions for (A) and (B). Binding to the oxoG lesion was observed for hOGG1$_{K249Q}$ ± Myc at comparable frequencies as with the wildtype protein (positions and Table 1). The specificities for oxoG binding were 294 ± 139 and 468 ± 197 for hOGG1$_{K249Q}$ and 438 ± 217 and 421 ± 153 for hOGG1$_{K249Q}$/Myc under oxidising and reducing conditions, respectively (standard deviations from triplicate experiments). Volume analyses (central columns) were consistent with a dimer for pre-oxidised hOGG1$_{K249Q}$ (~160 nm^3) and a monomer under reducing conditions (~100 nm^3). Volumes of DNA bound complexes in hOGG1$_{K249Q}$+Myc samples were ~240 nm^3 under oxidising as well as reducing conditions. Volume increases were Δ~60 nm^3 for the hOGG1 dimer compared to the monomer and Δ~80 nm^3 for the complex of hOGG1+Myc compared to hOGG1 dimer. DNA bend angle measurements (right columns) showed DNA bending of ~0°, ~35° and ~70° by hOGG1$_{K249Q}$ monomer and dimer (under reducing or oxidising conditions) as well as by hOGG1$_{K249Q}$/Myc complexes at non-specific DNA positions. At oxoG lesions, hOGG1$_{K249Q}$ in the absence of Myc showed comparable DNA bending as for non-specific DNA sites, however, hOGG1$_{K249Q}$/Myc complexes bound at oxoG displayed almost exclusively a single conformational state with DNA bend angle of ~20º.

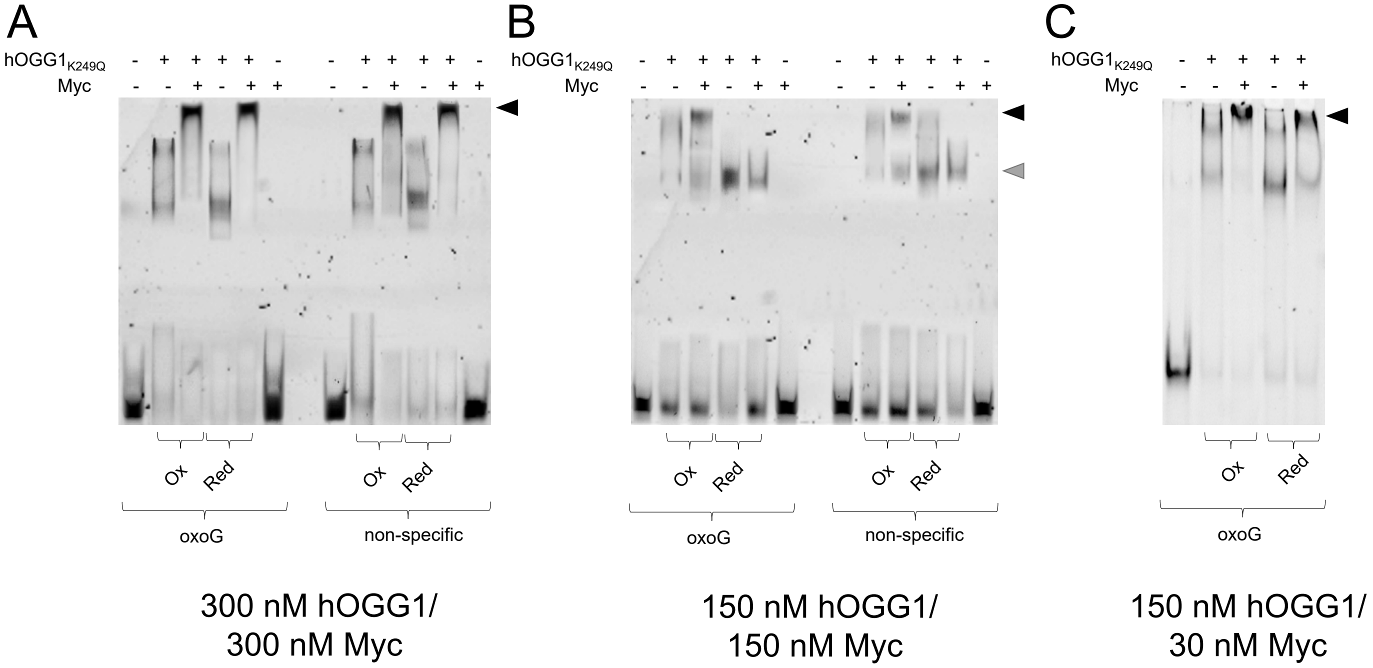

Fig. S10: EMSAs of hOGG1-Myc interaction on DNA. (A) Binding of hOGG1 (300 nM) to DNA substrate containing an oxoG lesion or undamaged (non-specific) DNA in the absence or presence of Myc (300 nM) shows comparable hOGG1-Myc complex formation on DNA under oxidising (Ox) as well as reducing (Red) conditions (black arrow). **(B)** Binding of hOGG1 (150 nM) to DNA substrate containing an oxoG lesion or undamaged (non-specific) DNA in the absence or presence of Myc (150 nM) shows hOGG1-Myc complex formation on DNA only for oxidised, dimeric hOGG1. At 150 nM, reduced monomeric hOGG1 is unable to support stable hOGG1-Myc complexes on the DNA. **(C)** Excess of hOGG1 over Myc supports hOGG1/Myc complex formation on oxoG DNA. At 150 nM hOGG1 and 30 nM Myc, both dimeric (oxidised) and monomeric (reduced) hOGG1 form complexes with Myc on oxoG containing DNA as can be seen from the shifts to slower electromobility species in the gel (black arrow). However, complex formation is clearly enhanced under the oxidising compared to reducing condition. To avoid interference with repair activity by hOGG1, the catalytically inactive K249Q variant of hOGG1 was used in all of these assays. Experiments were performed in triplicate with identical results.

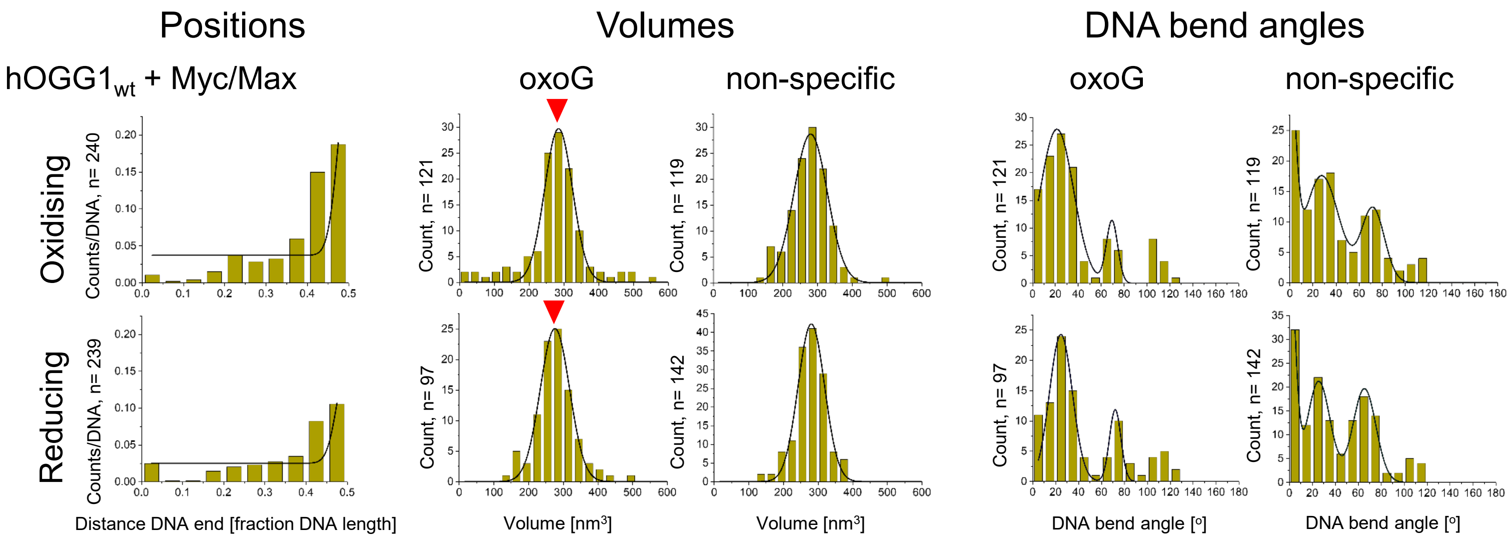

Fig. S11: AFM analyses of hOGG1/Myc/Max on oxoG-DNA. Pooled data from triplicate experiments on samples containing hOGG1$_{wt}$ (150 nM), Myc (30 nM), and Max (30 nM) with oxoG-DNA.under oxidising (top) or reducing conditions (bottom). Position distributions indicate strong recognition of the oxoG lesion by the complexes (specificities of 421 ± 252 and 456 ± 289 for oxidising and reducing conditions, respectively). Compared to hOGG1/Myc complexes (without Max), binding by hOGG1/Myc/Max to oxoG lesions was slightly enhanced under oxidising and decreased under reducing conditions (positions and Table 1). The volumes were increased (compared to without Max) by Δ~50 nm^3 to ~290 nm^3. The similar volume increase in the complexes as for hOGG1 dimers *versus* monomers (Δ~60 nm^3) is consistent with the molecular masses of the proteins. Max in our experiments has a molecular mass of 46 kDa (due to the large GST tag), which is similar to the size of hOGG1 (39 kDa) and smaller than the size of the GST-tagged Myc (76 kDa). DNA bending by the hOGG1/Myc/Max complex was comparable to bending by the hOGG1/Myc complex (see Figure 3B in main manuscript).

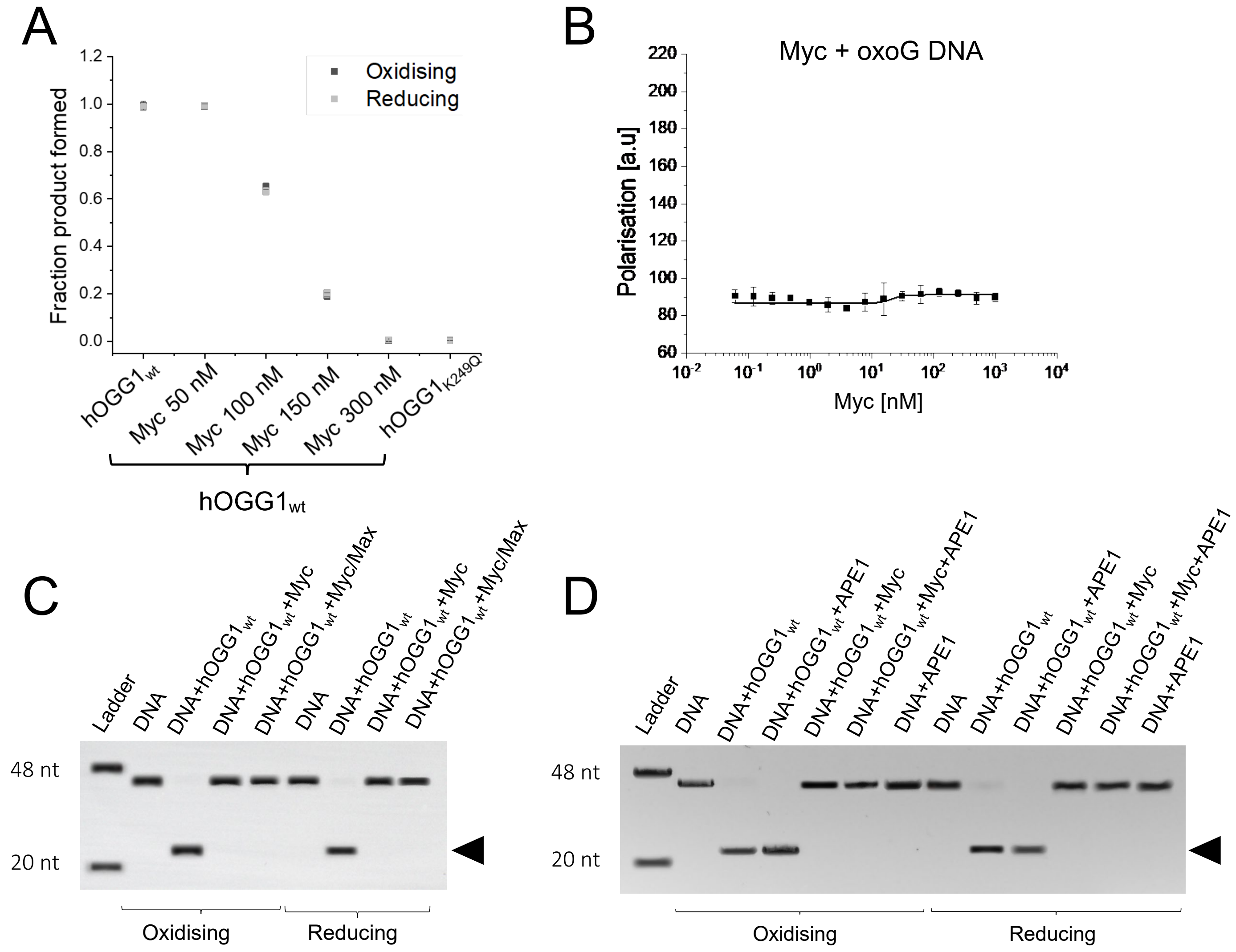

Fig. S12: Suppression of hOGG1 catalytic repair activity by c-Myc(/Max). (A) Quantification of the hOGG1 activity assay (Figure 4B) in the presence of increasing concentrations of c-Myc. **(B)** c-Myc does not bind to the oxoG containing DNA substrate in the assays under the conditions of our experiments, confirming that hOGG1 repair activity is not suppressed simply by c-Myc covering the oxoG lesion in the DNA. **(C)** Suppression of hOGG1 catalytic activity by c-Myc was independent of the absence or presence of Max. Samples of hOGG1 ± c-Myc ± Max were pre-incubated at 600 nM each for 15 min at 37°C in interaction buffer (20 mM Tris-HCl pH 7.6, 200 mM NaCl). Pre-incubated proteins were then mixed at 1:1 volume ratio with 40 nM oxoG + E-box containing DNA substrate (for final concentrations of 300 nM hOGG1, c-Myc, and Max, as indicated, and 20 nM DNA) for 30 min at 37°C either under oxidative (+ 5 µM H_2O_2) or reducing (+ 5 mM DTT) conditions. Samples were then incubated for 10 min with 0.5 N NaOH to induce strand breaks at product AP sites, followed by DNA strand separation by heating to 95°C for 10 min in the presence of 8 M urea, and application to 7 M urea gels. The arrow indicates product formation by hOGG1 catalytic activity in oxoG repair. **(D)** Suppression of hOGG1 activity by c-Myc was independent of APE1. Samples of hOGG1 ± c-Myc ± Max were pre-incubated as in (C) and then added to 20 nM of oxoG containing DNA substrate with or without APE1 at final concentrations of 300 nM hOGG1, c-Myc, Max, and APE1 (as indicated). Incubations were carried out as in (C). APE1 was obtained from Origene.

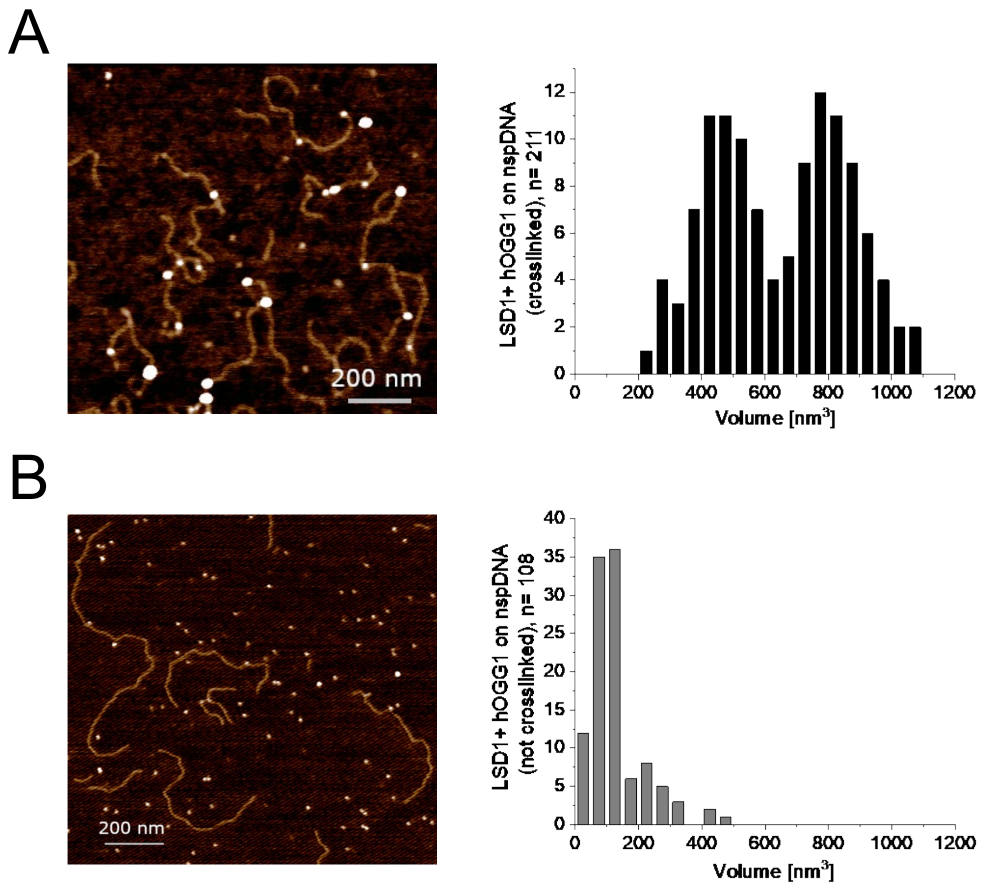

Fig. S13: AFM imaging of hOGG1-LSD1 on DNA requires crosslinking of complexes. (A) Representative AFM image of crosslinked hOGG1-LSD1 complexes on non-specific (nsp) DNA (left). Concentrations for sample deposition onto the mica substrate were 10 nM for LSD1 and hOGG1 each. Samples were incubated at 1 μM LSD1 and hOGG1 and 100 nM DNA at ambient temperature for 30 min, followed by 10 min crosslinking in 0.1% glutaraldehyde at ambient temperature, and immediate 100x dilution and deposition in AFM buffer. Volume distributions (right) indicate complexes of LSD1 and hOGG1 on the DNA. **(B)** Representative AFM image (left) of non-crosslinked samples of LSD1 (50 nM) and hOGG1 (150 nM) with nsp DNA (2 nM). Volumes (right) are consistent with hOGG1 in the absence of LSD1, indicating no stable complexes of LSD1 and hOGG1 on the DNA at these concentrations. Scale bars are 200 nm.
